# State of the health workforce in the WHO African Region: decade review of progress and opportunities for policy reforms and investments

**DOI:** 10.1136/bmjgh-2024-015952

**Published:** 2024-11-25

**Authors:** James Avoka Asamani, Kouadjo San Boris Bediakon, Mathieu Boniol, Joseph Kyalo Munga’tu, Christmal Dela Christmals, Sunny C. Okoroafor, Adam Ahmat, Maritza Titus, Jean Benard Moussounda, Hillary Kipruto, Kasonde Mwinga, Joseph Waogodo Cabore, Matshidiso Rebecca Moeti

**Affiliations:** 1 Health Workforce Unit, Universal Health Coverage Life Course, World Health Organization Regional Office for Africa, Brazzaville, Congo; 2 Centre for Health Professions Education, North-West University - Potchefstroom Campus, Potchefstroom, South Africa; 3 Health Workforce, World Health Organization, Geneva, Switzerland; 4 Actuarial Science, Jomo Kenyatta University of Agriculture and Technology, Nairobi, Kenya; 5 Inter-Country Support Team for Eastern and Southern Africa, World Health Organization Regional Office for Africa, Harare, Kenya; 6 Universal Health Coverage Life Course Cluster, World Health Organization Regional Office for Africa, Brazzaville, Congo; 7 Programme Management, World Health Organization Regional Office for Africa, Brazzaville, Congo; 8 World Health Organization, Regional Office for Africa, Brazzaville, Congo

**Keywords:** Review, Health policies and all other topics, Health economics, Public Health, Health services research

## Abstract

**ABSTRACT:**

**Introduction:**

An adequate health workforce is one of the cornerstones of a healthy nation. Over the last two decades, Africa has gained momentum in mitigating critical health workforce gaps, but urgent actions are still needed to accelerate progress towards universal health coverage and ensuring health security. This analysis provides an overview of the health workforce in the WHO African Region for the last decade.

**Methods:**

Data were extracted and triangulated from the National Health Workforce Accounts (NHWA), health labour market analyses, countries’ human resources for health (HRH) profiles, HRH strategic plans and annual reports. A descriptive analysis of health worker stock, training capacity and unemployment levels was undertaken. The density of health workers was calculated per 10 000 population for each country and examined by occupational groups and income levels of the countries to provide a more comprehensive understanding of the health workforce dynamics.

**Results:**

The stock of the health workforce progressively increased from 1.6 million in 2013 to 4.3 million in 2018 and 5.1 million in 2022. The stock of doctors, nurses, midwives, dentists and pharmacists was 2.6 million in 2022, representing a threefold increase over 10 years, with an annual growth rate of 13%. The density of these five health workforce occupations grew by 1.9% per annum between 2018 and 2022, from 11.14 per 10 000 in 2013 to 26.82 per 10 000 in 2022. The health professions education capacity in the region increased by 70%, with the annual education output growing from 148 357 graduates in 2018 to over 255 000 in 2022. The comprehensiveness of the findings can be attributed to improvement in health workforce data availability and quality as more countries implement the NHWA. The improvements in the health workforce in the region are also partly attributable to increasing investments in the capacity of health professions education institutions to produce more health workers, and use of evidence in planning, decision-making and high-level advocacy at various levels to invest in health workers.

**Conclusion:**

This study provides crucial insights for policy reforms and investments to enhance the health workforce, which is essential to achieving universal health coverage and ensuring health security. While progress is notable, countries with unique challenges need targeted analyses and continuous support to develop the necessary number and skills of health workers in the African region.

**WHAT IS ALREADY KNOWN ON THIS TOPIC**

- The African region faces multifaceted health workforce challenges.
- Over the years, countries in the region have implemented strategies to mitigate critical health workforce shortages and address identified challenges.

**WHAT THIS STUDY ADDS**

- The study presents up-to-date information on health workforce density in the African region, capacity for training, unemployment levels and migration as of 2022.
- In 2022, there were 27 doctors, nurses, midwives, dentists and pharmacists (Sustainable Development Goal 3c occupations) per 10 000 people in the region, a 2.5-fold improvement compared with 11 per 10 000 people in 2013.
- Population growth is outpacing the growth of health workers, with 37 countries showing a positive trajectory in increasing health worker head count between 2018 and 2022, but the workforce density per 10 000 population increasing in only 29 countries, which illustrates that populations are growing faster than the rate of workforce development and marginally due to outmigration.
- The African region produces at least 255 000 skilled health workers per year, which is equivalent to one health worker trained for every 10 workers already employed, but almost one in three skilled health workers (27%; 95% CI 14%, 39%) in the region are paradoxically unemployed despite a 6.1 million need-based shortage at the frontlines of health service delivery.

**HOW THIS STUDY MIGHT AFFECT RESEARCH, PRACTICE OR POLICY**

- The findings should shape policy and investment dialogues to strengthen the health workforce across the region, achieve universal health coverage and ensure health security.
- While countries continue to face similar challenges, they have different abilities in terms of education and training, and as such it is essential to tailor solutions to the contextual realities of a country.

## Introduction

 The Global Strategy on Human Resources for Health: Workforce 2030^1^ aims to accelerate progress towards achieving universal health coverage (UHC) and the Sustainable Development Goals (SDGs) by ensuring equitable access to health workers within a strengthened health system. The strategy emphasises the need to optimise the existing health workforce and to continue making adequate investments in education, recruitment, development, motivation and retention, as well as to ensure adequate availability and fair distribution of health workers with an appropriate skill mix.^1^

The WHO African Region’s framework for implementing the Global Strategy on Human Resources for Health^2^ further contextualised various global strategies and initiatives by incorporating the region’s social, political, economic and cultural dynamics and health worker projections.^2^ The development and implementation of this framework recognised the peculiar dynamics within the region, including that Africa has about 10% of the global population and contributes to 25% of the world disease burden, with only about 4% of the global health workforce to provide needed care.^3 4^ Despite the African region suffering many setbacks in providing the needed essential healthcare services,^5^,^8^ modest progress has been made in improving life expectancy, partly contributed by the increase in the production of health workers of various cadres.^9 10^

Evidence has shown that increasing investments in the health workforce has the triple effect of improving healthcare outcomes, ensuring global health security and stimulating economic growth.^11^,^13^ Specifically, every US$1 dollar invested in the general health workforce results in a return on investment of about US$9, and an even higher return on investment for community health workers.^14 15^

The recent drive to build better health systems and expedite the achievement of the SDGs before 2030 necessitates a thorough review of the performance of the health workforce. To better track progress and signpost policy and strategic actions towards the 2030 targets for the SDGs, evidence on the state of the health workforce in the African region is urgently needed. The need for contemporary evidence on the health workforce, particularly in the aftermath of the global COVID-19 crisis, informed our analysis, which provides insights into the progress made in the stock and density of the health workforce in the WHO African Region between 2013 and 2022. We also provide an overview of the capacity of health training institutions to produce health workers and the health worker employment dynamics.

## Methods

Data were primarily extracted and triangulated from multiple sources. Data on stock (disaggregated by country, gender and age) and training capacity were obtained from the National Health Workforce Accounts (NHWA) and supplemented with data obtained from health labour market analysis (HLMA) reports.

The NHWA is a system whereby countries progressively improve the availability, quality and use of health workforce data by tracking a set of indicators in order to support the attainment of the SDGs, UHC and other national health goals. The NHWA provides a standardised framework for countries to report comparable data using standard definitions of occupations aligned with the International Standard Classification of Occupations 2008 and recommended sources of data for various health workforce indicators.^16^

**Figure 1:**
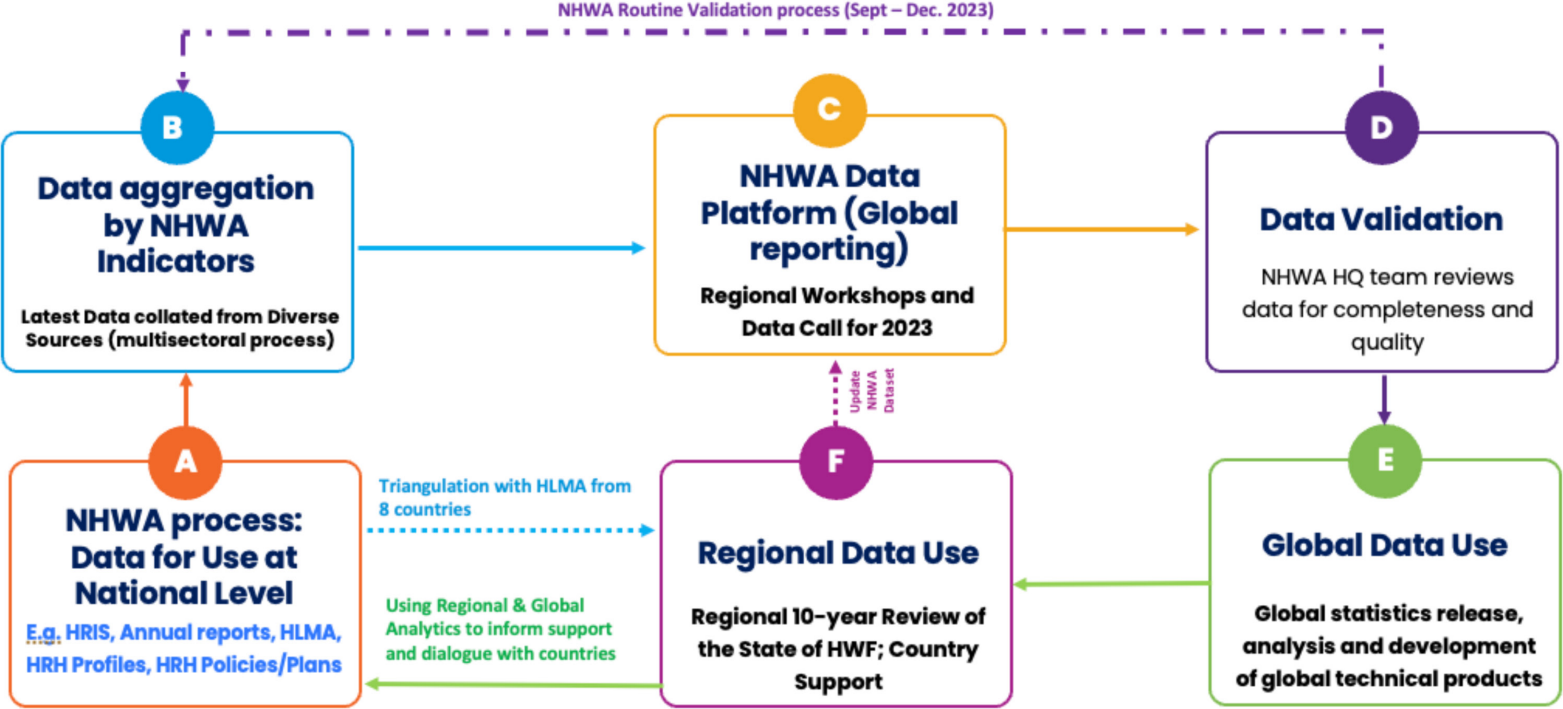
Data triangulation approach. HQ, Headquarters; HLMA, health labour market analysis; HRH, human resources for health; HWF, health workforce; HRIS, Human Resources Information System; NHWA, National Health Workforce Accounts.

Each country has an NHWA focal point that coordinates a process of national dialogue, data collation from stakeholders and validation of the data in line with the country’s needs, and then reports the data to the WHO through the annual reporting cycle via a dedicated NHWA data platform. At the country level, typical stakeholders involved in contributing data are the ministries of health, education, labour, finance, national statistics authorities, health professional regulatory bodies, labour unions and the private sector, where they are organised as a body. Data submitted to the WHO through the NHWA data platform undergo a series of quality checks, and the country’s NHWA focal points usually provide explanations or corrections as appropriate before the submitted data are released as official statistics (see figure 1). The NHWA data portal serves as the primary source of data for global monitoring of health workforce indicators and targets outlined in the Global Strategy on Human Resources for Health: Workforce 2030 and SDG 3c.

Following 5 years of implementation, the WHO introduced the second version of the NHWA, which has further standardised health workforce data definitions and reporting processes. In July 2023, the WHO Regional Office for Africa convened 113 national NHWA focal points and experts from WHO country offices across countries in the African region for a 5-day workshop to strengthen data availability and quality for this analysis. The objective was to harmonise the understanding of the second version of the NHWA and review and update each country’s data. As a result, 45 out of 47 countries (95.7%) in the WHO African Region successfully submitted their latest health workforce data, covering up to 2022, through the NHWA platform.

### Data collection and triangulation procedure

A three-step approach was adopted to triangulate and address data quality issues: (1) identification of data sources, (2) selection of data points and (3) adjustments for the private sector.

#### Data sources

We predominantly relied on the NHWA,^16^ which can be accessed via the NHWA data platform (https://apps.who.int/nhwaportal/Home/Index), as the institutionalised mechanism for countries to publicly share their health workforce statistics. Wherever the NHWA had missing data points, data from HLMA reports, health workforce country profiles, health workforce strategic plans and annual reports of professional regulatory councils were used (see table 1).

**Table 1:** Data sources for each country

Country	Country code	Data sources
Algeria	DZA	NHWA data set
Angola	AGO	NHWA data set
Benin	BEN	NHWA data set
Botswana	BWA	NHWA data set
Burkina Faso	BFA	NHWA data set
Burundi	BDI	NHWA data set
Cabo Verde	CPV	NHWA data set
Cameroon	CMR	NHWA data set
Central African Republic	CAF	NHWA data set
Chad	TCD	NHWA data set
Comoros	COM	NHWA data set
Congo	COG	NHWA data set
Côte d’Ivoire	CIV	NHWA data set
Democratic Republic of the Congo	COD	NHWA data set
Equatorial Guinea	GNQ	NHWA data set
Eritrea	ERI	NHWA data set
Eswatini	SWZ	NHWA data set and HLMA 2023
Ethiopia	ETH	NHWA data set and HLMA 2020
Gabon	GAB	NHWA data set
The Gambia	GMB	NHWA data set
Ghana	GHA	NHWA data set and HLMA 2023
Guinea	GIN	NHWA data set
Guinea-Bissau	GNB	NHWA data set
Kenya	KEN	NHWA data set and HLMA 2023
Lesotho	LSO	NHWA data set and HLMA 2021
Liberia	LBR	NHWA data set
Madagascar	MDG	NHWA data set
Malawi	MWI	NHWA data set
Mali	MLI	NHWA data set and HLMA 2023
Mauritania	MRT	NHWA data set
Mauritius	MUS	NHWA data set
Mozambique	MOZ	NHWA data set and HLMA 2023
Namibia	NAM	NHWA data set
Niger	NER	NHWA data set
Nigeria	NGA	NHWA data set and HRH country profile
Rwanda	RWA	NHWA data set and HLMA 2019
São Tomé and Príncipe	STP	NHWA data set
Senegal	SEN	NHWA data set
Seychelles	SYC	NHWA data set
Sierra Leone	SLE	NHWA data set and HLMA 2019
South Africa	ZAF	NHWA data set and Nursing Council Annual Report, Health Professions Council 2022
South Sudan	SSD	NHWA data set
Tanzania	TZA	NHWA data set
Togo	TGO	NHWA data set
Uganda	UGA	NHWA data set and HLMA 2023
Zambia	ZMB	NHWA data set and HLMA 2023
Zimbabwe	ZWE	NHWA data set and HLMA 2022

HLMA, health labour market analysis; HRH, human resources for health; NHWA, National Health Workforce Accounts.

#### Selection of data points for 2013, 2018 and 2022

The data set revealed that there were data gaps for some years, leading to breaks in trends. As a result, time series analysis was not feasible; therefore, cross-sectional comparisons of three time points of 2013, 2018 and 2022 were used for the analysis. Data reported for 2013 or the nearest year between 2012 and 2014 were used for the 2013 estimate. Similarly, data reported for 2018 or the nearest year between 2016 and 2019 were used for the 2018 estimate. For the 2022 estimate, the latest reported data were used, of which 46 countries reported in 2022 (see table 2).

**Table 2:** Data availability for the listed occupations across 47 countries for 2013, 2018 and 2022

NHWA Code	ISCO-08 code	Occupation	Countries with 2013 data (n)	Countries with 2018 data (n)	Countries with 2022 data (n)
1	221	Medical doctors	32	47	47
1.1	2211	Generalist medical practitioners	5	47	47
1.2	2212	Specialist medical practitioners	8	45	47
2		Nursing personnel	37	47	47
2.1	2221	Nursing professionals	16	47	47
2.2	3221	Nursing associate professionals	16	37	39
3		Midwifery personnel	34	42	44
3.1	2222	Midwifery professionals	30	41	42
3.2	3222	Midwifery associate professionals	14	26	27
4	2230 and 3230	Traditional and complementary medicine practitioners	7	21	26
5	2240	Paramedical practitioners	6	30	33
6	2261	Dentists	21	46	46
7	2262	Pharmacists	27	47	47
8	2263 and 3257	Environmental and occupational health hygiene workers	26	44	47
9	2264 and 3255	Physiotherapists and physiotherapy assistants	11	36	40
10	2265	Dietitians and nutritionists	21	34	40
11	2266	Audiologists and speech therapists	1	8	20
12	2267 and 3254	Optometrists and ophthalmic opticians	6	32	34
13	2634	Psychologists		12	23
14	2635 and 3412	Social workers	7	11	18
15	3211	Medical imaging and therapeutic equipment technicians	13	42	42
16	3212	Medical and pathology laboratory technicians	14	42	43
17	3213	Pharmaceutical technicians and assistants	12	40	44
18	3214	Medical and dental prosthetic technicians	8	34	38
19	3251	Dental assistants and therapists	6	35	38
20	3252	Medical records and health information technicians	1	32	33
21	3253	Community health workers	9	36	40
22	3256	Medical assistants		26	29
23	5321, 5322 and 5329	Personal care workers in health service		32	35
24	1342	Managerial staff	1	36	38
25		Medical and pathology laboratory scientists	9	46	47
26		Other non-medical professional staff		34	36
27		Other non-medical support staff		29	34

ISCO-08, International Standard Classification of Occupations 2008; NHWA, National Health Workforce Account.

#### Adjustment for private sector contribution

It became apparent that, for some occupations, the 2022 data reported through the NHWA focused mainly on the public sector. This was clear when the 2022 data were compared with the 2018 survey data point. To accommodate the private sector’s contribution to the health workforce, proportions of the private sector from the 2018 regional survey^5 17^ were used to adjust the 2022 reported values, where applicable. Overall, 31% of the 1258 data points from 2022 were adjusted, resulting in a 5% (245 866) increase in the estimated number of health workers. The adjustment affected an average of 8 of the 33 occupations across all countries (see tables3  4).

**Table 3:** Number of occupations (out of 33) adjusted across countries

S/N	Country	Occupations not adjusted (n)	Occupations adjusted (n)	Occupations reported (n)	Occupations adjusted (%)
1	Algeria	21	9	30	30
2	Angola	16	16	32	50
3	Benin	24	2	26	8
4	Botswana	14	11	25	44
5	Burkina Faso	21	11	32	34
6	Burundi	18	9	27	33
7	Cameroon	30	3	33	9
8	Cabo Verde	20	9	29	31
9	Central African Republic	16	11	27	41
10	Chad	19	11	30	37
11	Comoros	14	3	17	18
12	Congo	15	6	21	29
13	Côte d’Ivoire	19	10	29	34
14	Democratic Republic of the Congo	14	7	21	33
15	Equatorial Guinea	15	4	19	21
16	Eritrea	13	10	23	43
17	Eswatini	18	14	32	44
18	Ethiopia	26	2	28	7
19	Gabon	15	18	33	55
20	The Gambia	14	11	25	44
21	Ghana	24	7	31	23
22	Guinea	13	7	20	35
23	Guinea-Bissau	14	15	29	52
24	Kenya	21	4	25	16
25	Lesotho	17	12	29	41
26	Liberia	17	7	24	29
27	Madagascar	21	4	25	16
28	Malawi	19	10	29	34
29	Mali	20	3	23	13
30	Mauritania	20	6	26	23
31	Mauritius	20	10	30	33
32	Mozambique	23	8	31	26
33	Namibia	17	14	31	45
34	Niger	18	8	26	31
35	Nigeria	18	8	26	31
36	Rwanda	19	1	20	5
37	São Tomé and Príncipe	11	3	14	21
38	Senegal	14	18	32	56
39	Seychelles	18	6	24	25
40	Sierra Leone	17	9	26	35
41	South Africa	18	10	28	36
42	South Sudan	23	4	27	15
43	Tanzania	15	12	27	44
44	Togo	19	13	32	41
45	Uganda	20	4	24	17
46	Zambia	26	1	27	4
47	Zimbabwe	28	5	33	15

**Table 4:** Occupations for which adjustments were made

S/N	Occupation	Countries not adjusted (n)	Countries adjusted (n)	Countries that reported (n)	Countries adjusted (%)
1	Dental assistants and therapists	22	16	38	42
2	Dentists	22	24	46	52
3	Generalist medical practitioners	33	14	47	30
4	Medical doctors	28	19	47	40
5	Midwifery associate professionals	22	5	27	19
6	Midwifery personnel	31	13	44	30
7	Midwifery professionals	26	16	42	38
8	Nursing associate professionals	26	13	39	33
9	Nursing personnel	31	16	47	34
10	Nursing professionals	27	20	47	43
11	Pharmaceutical technicians and assistants	35	9	44	20
12	Pharmacists	20	27	47	57
13	Psychologists	20	3	23	13
14	Specialist medical practitioners	22	25	47	53
15	Audiologists and speech therapists	16	4	20	20
16	Community health workers	36	4	40	10
17	Dietitians and nutritionists	30	10	40	25
18	Environmental and occupational health hygiene workers	34	13	47	28
19	Managerial staff	35	3	38	8
20	Medical and dental prosthetic technicians	25	13	38	34
21	Medical and pathology laboratory scientists	31	16	47	34
22	Medical and pathology laboratory technicians	28	15	43	35
23	Medical assistants	22	7	29	24
24	Medical imaging and therapeutic equipment technicians	33	9	42	21
25	Medical records and health information technicians	27	6	33	18
26	Optometrists and ophthalmic opticians	20	14	34	41
27	Other non-medical professional staff	26	10	36	28
28	Other non-medical support staff	28	6	34	18
29	Paramedical practitioners	23	10	33	30
30	Personal care workers in health service	28	7	35	20
31	Physiotherapists and physiotherapy assistants	28	12	40	30
32	Social workers	15	3	18	17
33	Traditional and complementary medicine practitioners	22	4	26	15

### Data analysis

#### Stock and density

A descriptive analysis of the stock data was undertaken in head counts. The density of health workers in each country within the region was calculated and expressed per 10 000 population. This metric is essential in comparing the available health workers to the population size, is a key indicator of the availability of health workers and connotes the capacity of a health system to deliver services.

The analysis was disaggregated by occupational groups and income levels to provide a more comprehensive understanding of the health workforce composition and dynamics. This process facilitated the identification of trends among various categories of health workers, especially the SDG 3c.1 tracer occupations (medical doctors, nurses, midwives, pharmacists and dentists).

#### Training capacities

A descriptive analysis of the training and education capacities and outputs in the region was undertaken. The relationship between training outputs and the density of available stock per population was examined to determine the relative adequacy or otherwise of the outputs from the education pipeline.

### Health workforce employment

A descriptive analysis of unemployment levels in terms of the proportion of unemployed health workers was conducted from a subset of 10 countries that conducted HLMAs between 2019 and 2023. These 10 countries used a similar methodology in their HLMAs.

### Patient and public involvement in the study

The study was based on publicly available data through a routine data-sharing process between Member States and WHO. Patients and the public were not involved in this study’s design, conduct or reporting.

## Results

### Data availability by occupation

**Figure 2:**
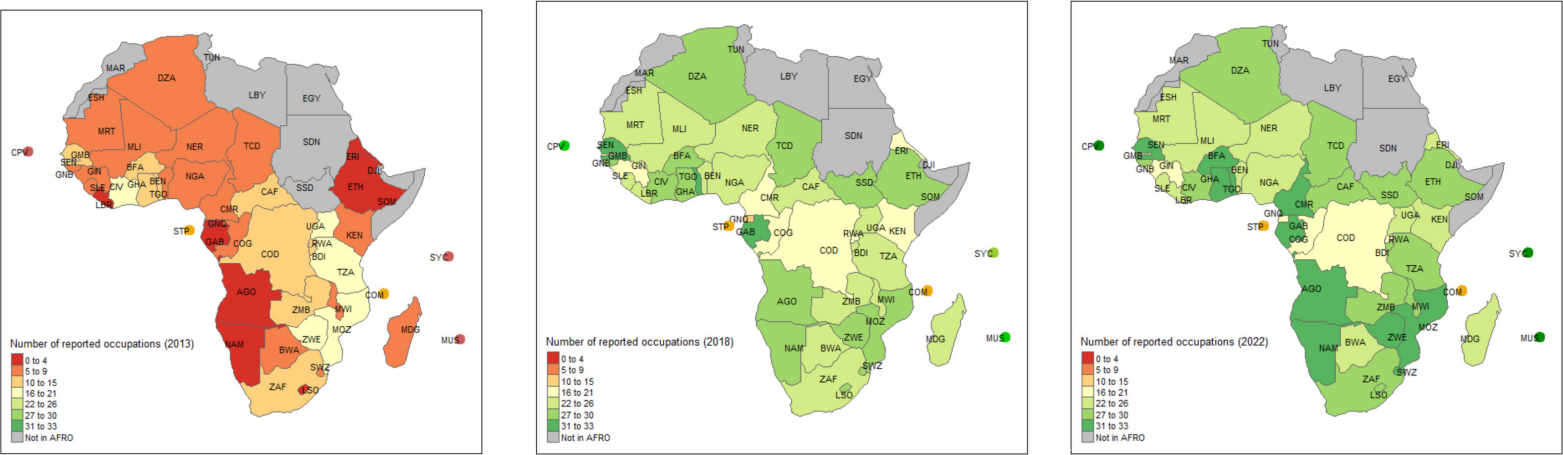
Data availability in the African region for the years 2013, 2018 and 2022. AFRO, African Region; country codes are defined in table 2.

Reporting by occupation improved between 2013 and 2022. In 2013, data on only 26% of the occupations were reported across all countries, which improved to 75% in 2018 and 81% in 2022. None of the countries in the African region reported data on all the occupations in 2013. In 2018, only one country (Gabon) reported all the occupations, and in 2022 three countries (Cameroon, Gabon and Zimbabwe) reported data on all the occupations (figure 2).

### Stock and density of health workers

**Figure 3:**
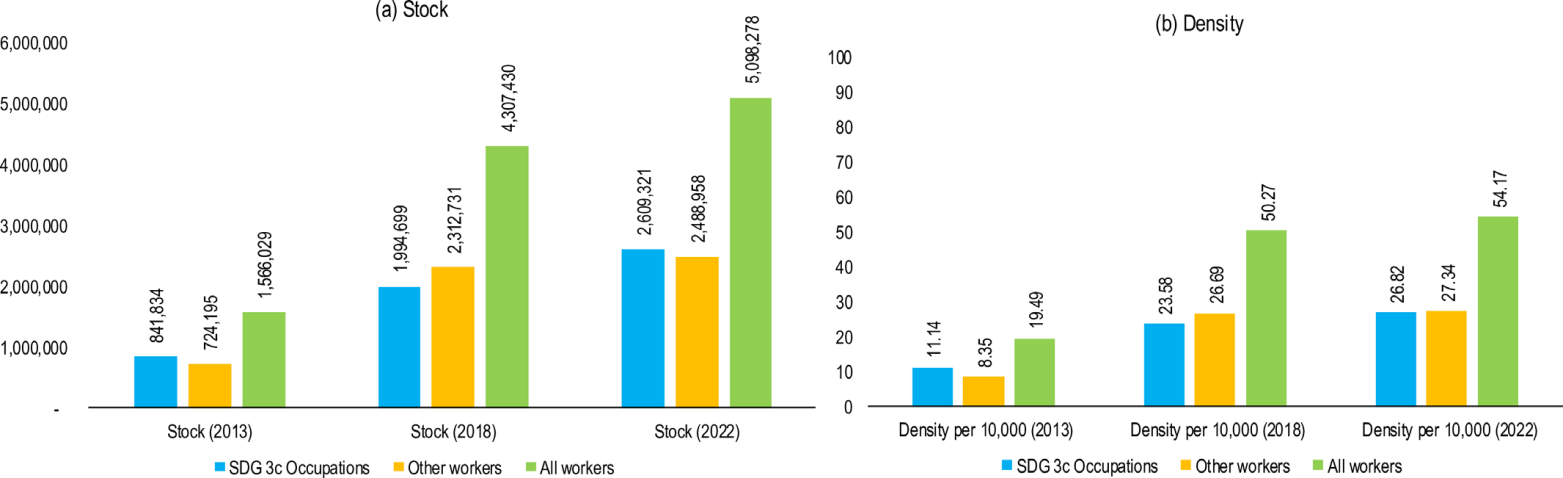
Trends in stock (**a**) and density (**b**) between 2013 and 2022. SDG, Sustainable Development Goal.

In 2022, there were 5.1 million health workers of any kind reported by countries in the WHO African Region compared with 4.3 million in 2018 and 1.6 million in 2013 (figure 3a). The health workforce stock increased by almost threefold between 2013 and 2022. Between 2018 and 2022, when data completeness and quality had improved, the overall stock of the health workforce grew by 4.3% per annum across all professions and 6.9% when the SDG 3c.1 tracer occupations (medical doctors, nurses, midwives, pharmacists and dentists) were considered. Comparing 2013 and 2022, the growth rate was 14% every year for all the occupations, with the SDG 3.c occupations growing at 13% and other workers at 14.7% per year between 2013 and 2022.

The density of all health workers is shown in figure 3b. In 2022, there were 27 doctors, nurses, midwives, dentists and pharmacists per 10 000 people in the region. This represents a 14% improvement compared with 2018 and more than doubled when compared with 2013. However, this varies widely from low-density countries, such as Niger (2.36), Central African Republic (2.41) and Chad (3.58), to relatively high-density countries, such as Namibia (72.5), South Africa (78.19) and Seychelles (242.01). More details on the specific occupations are found in online supplemental material 1. Full data tables are provided in online supplemental material 2.

**Figure 4:**
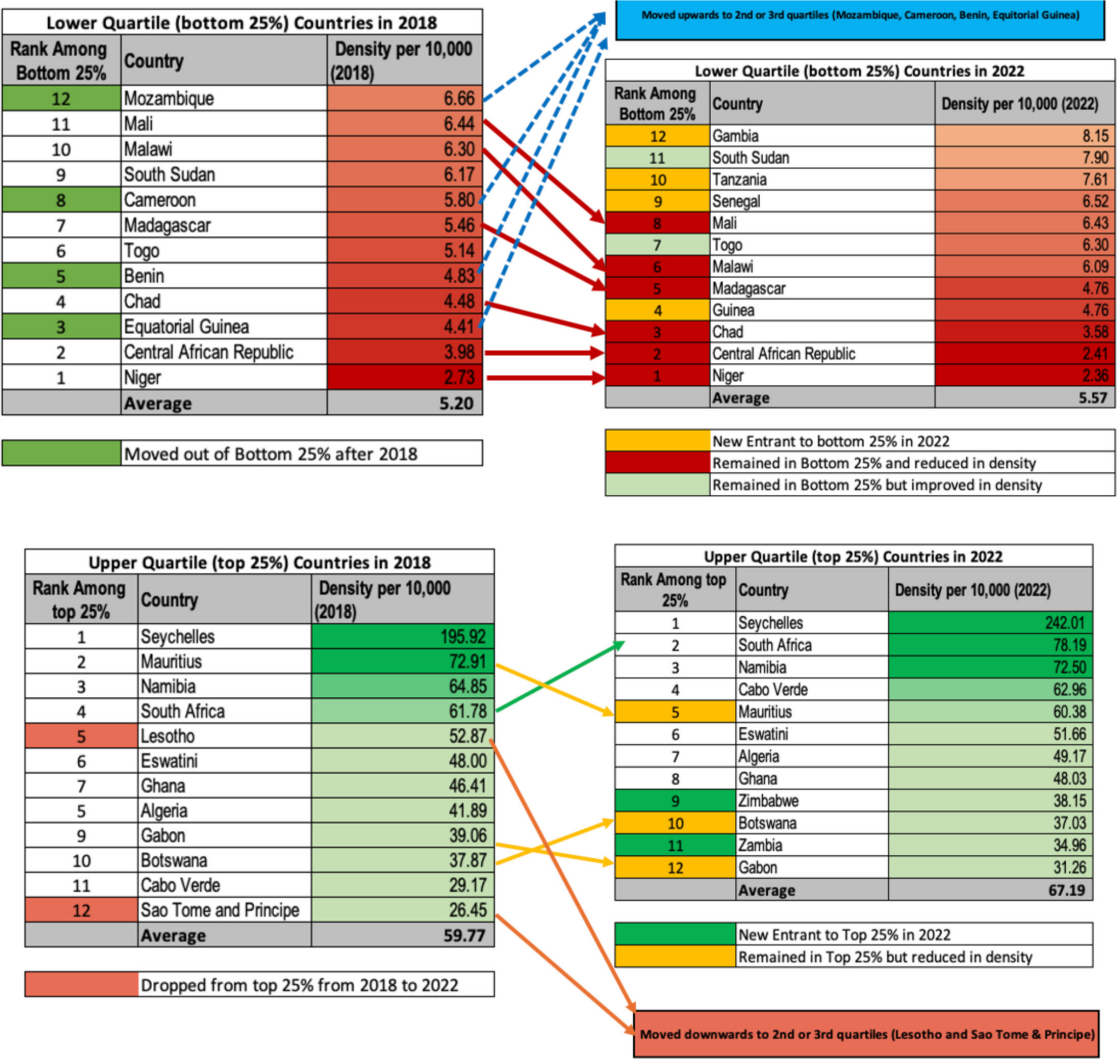
Changes in SDG 3c densities for the upper and lower quartiles. SDG, Sustainable Development Goal.

The 12 countries with the highest densities had 12 times more health workers per 10 000 population than the 12 countries with the lowest densities (figure 4). The upper 25% of the countries or the upper quartile (12 countries) with the highest densities have an average of 67.19 doctors, nurses, midwives, dentists and pharmacists per 10 000 population compared with a density of 5.57 for countries in the lower quartile (12 countries). Although the countries have improved in their densities, the level of disparity has not shown signs of improvement since 2018.

The density of all the occupations grew by 1.9% per annum between 2018 and 2022, with SDG 3c.1 tracer occupations growing by 3.2% and the other health workforce by 0.6% per annum in the same period. Countries with high SDG 3c.1 occupation densities as of 2022 were Algeria, Botswana, Cabo Verde, Eswatini, Gabon, Ghana, Mauritius, Namibia, Seychelles, South Africa and Zimbabwe. Countries with the least SDG 3c.1 tracer occupation densities were Central African Republic, Chad, Guinea, Madagascar, Malawi and Niger (figure 4).

While 79% of the countries showed a positive trajectory in increasing their stock between 2018 and 2022, when the population is considered, 62% of the countries improved their densities, while 38% did not show improvement. Of the 47 countries, 79% improved the head count of the health workforce between 2018 and 2022, while in 21% (10 countries) the stock did not improve. In eight countries, or 17% of the region (Botswana, Burundi, Democratic Republic of Congo (DRC), Malawi, Mali, Niger, Rwanda and Senegal), the stock was increased, but was outpaced by population growth. For 21% of the countries, the stock of health workers and the density reduced between 2018 and 2022. These are Central African Republic, Chad, Eritrea, Gabon, The Gambia, Guinea, Lesotho, Liberia, Madagascar and Mauritius.

There were 2.6 million doctors, nurses, midwives, dentists and pharmacists in the African region, a threefold increase over the 10-year period and an annual growth rate of 13%. Of the total number of doctors, nurses, midwives, dentists and pharmacists in the region, one in two were in lower-middle-income countries and one in three in low-income countries.

About 50% of the stock of doctors, nurses, midwives, dentists and pharmacists in the region were in lower-middle-income countries, but this is a 6% reduction in their share of the 2013 stock in the region. Twenty-seven per cent were in low-income countries, which has not changed since 2013. The high-income and upper-middle-income countries increased their share of stock of doctors, nurses, midwives, dentists and pharmacists in the same period, from 13% to 20% in 2022.

**Figure 5:**
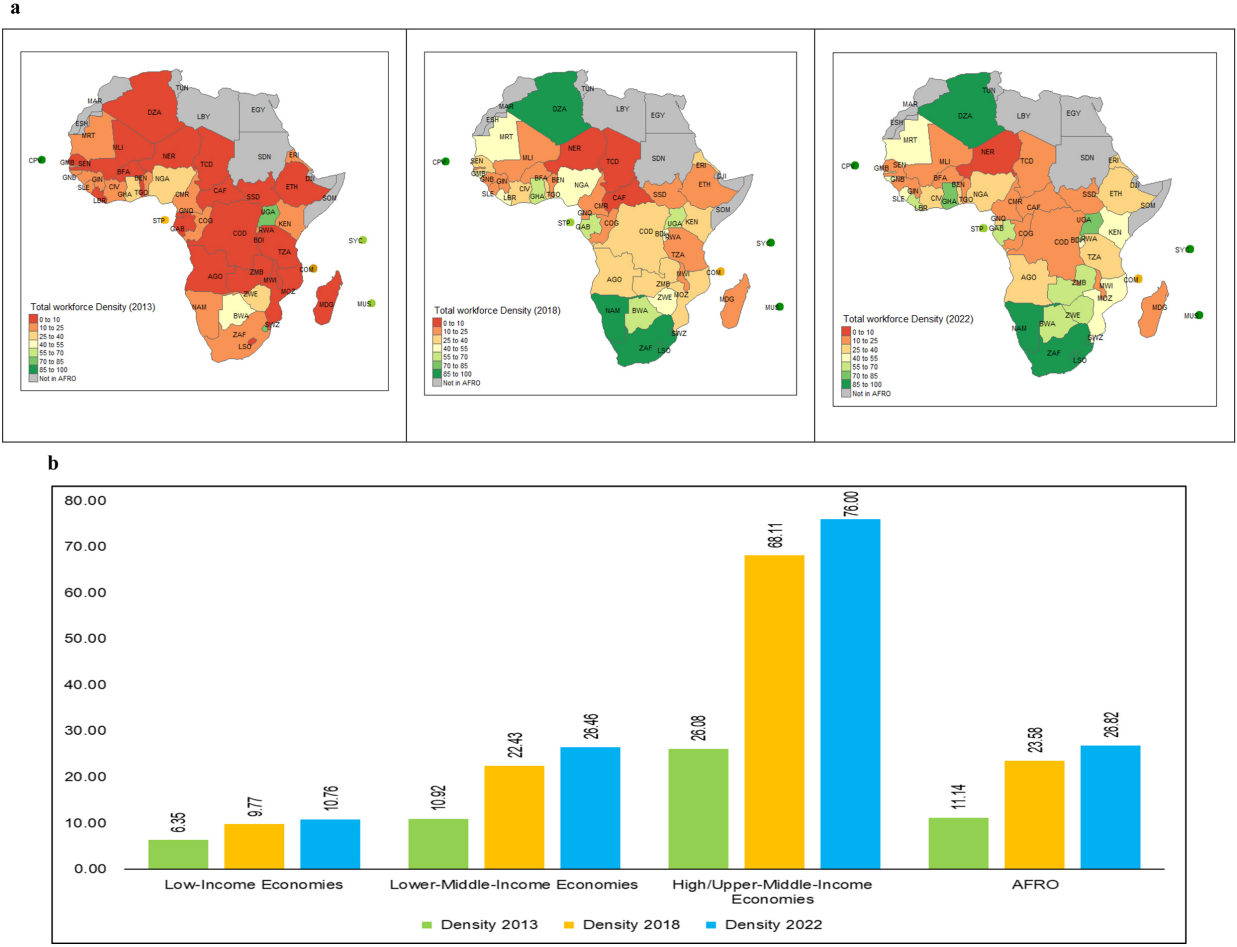
(**a**) Trend in SDG 3c tracer occupation density per 10 000 population between 2013 and 2022. (**b**) Density of SDG 3c occupations per 10 000 population in the region with income levels. SDG, Sustainable Development Goal.

Across all the income groups, density has grown significantly, with the overall density doubling from 11.14 per 10 000 in 2013 to 26.82 per 10 000 in 2022. High-income and upper-middle-income countries tripled their average workforce density from 26.08 to 76, followed by lower-middle-income countries, which more than doubled their densities from 10.92 in 2013 to 26.46 in 2022. Although low-income countries also recorded improvements, it was at a slower pace of 68%, increasing the density from 6.35 to 10.76 in the same period (figure 5).

**Figure 6:**
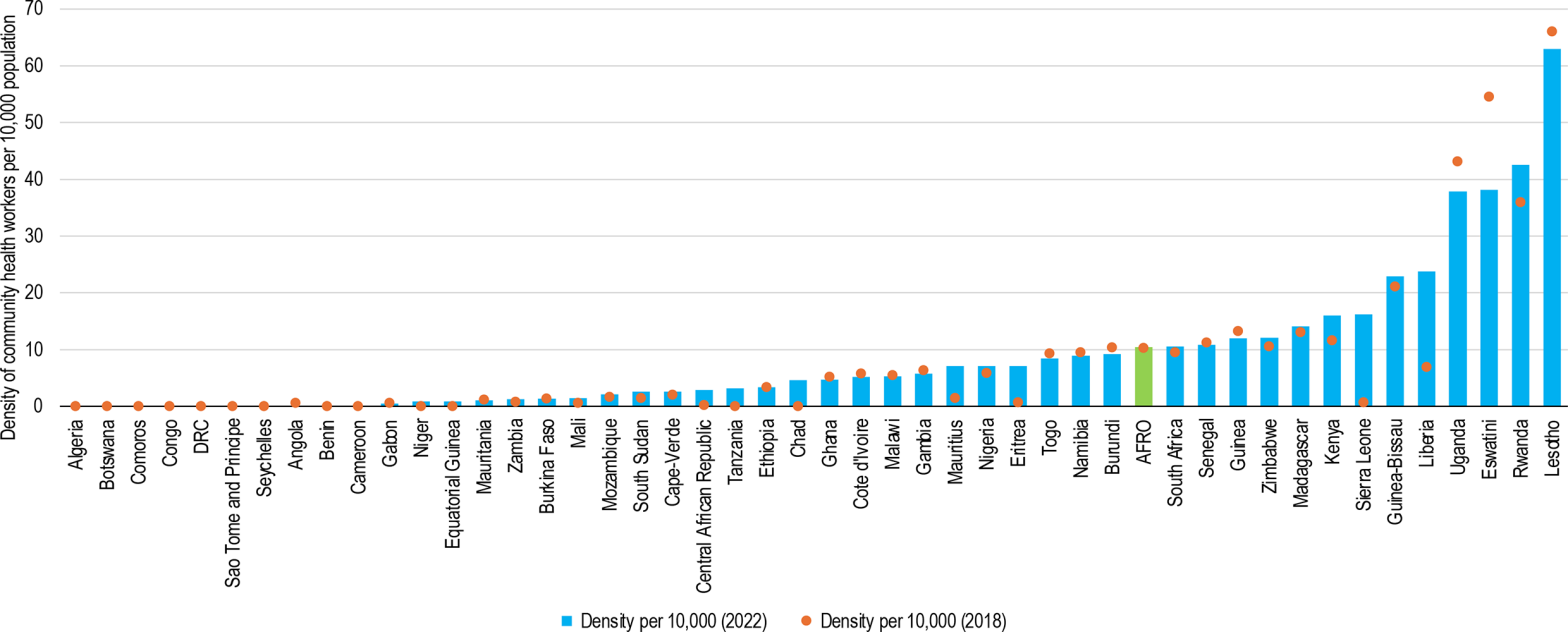
Density of community health workers in 2018 and 2022. Note that countries with zero entries did not report on community health workers. DRC, Democratic Republic of Congo.

The stock of community health workers was highly influenced by the number of countries that reported data (9, 36 and 40 countries in 2013, 2018 and 2022, respectively) out of the 47 countries in the African region (figure 6). Among the reporting countries, the number of community health workers number grew from 213 167 in 2013 to 850 462 in 2022. Correspondingly, the density per 10 000 population rose from 8.17 in 2013 to 10.43 in 2022.

### Demographic characteristics of health workers

#### Distribution by sex

**Figure 7:**
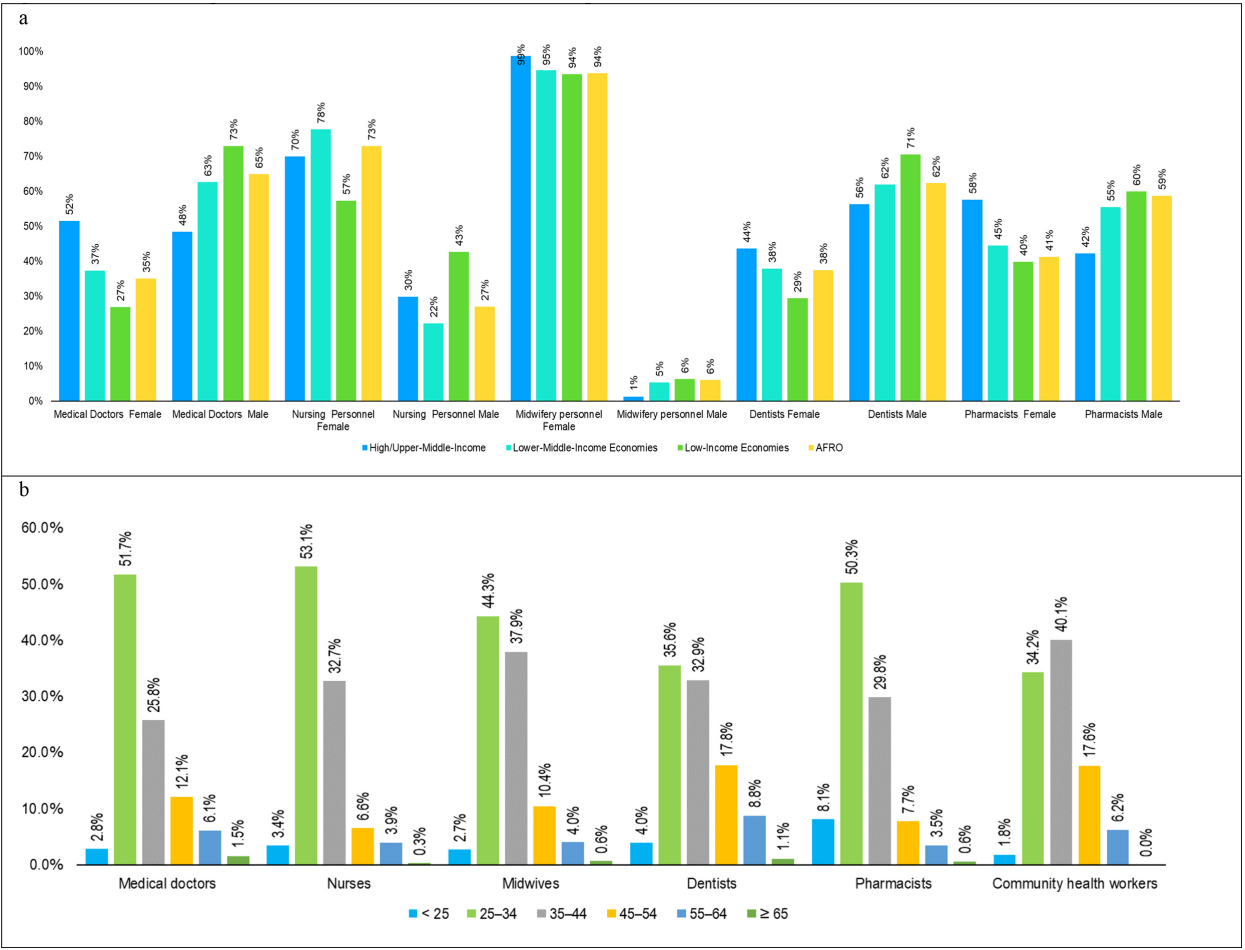
Gender and age distribution of the SDG 3c tracer occupations with the country’s income level. (**a**) Gender distribution of the SDG 3c tracer occupations. (**b**) Age distribution of selected occupations in the region. SDG, Sustainable Development Goal.

Data from 36 countries were comparable and analysed for the health workforce composition with respect to sex. The SDG 3c.1 tracer occupations in the African region have more female workers (72%) than male workers (28%). However, this is driven more by midwives (94%), community health workers (79%) and nurses (73%), who are more feminised, while pharmacy, dentistry and medicine are predominantly male (see figure 7a).

About 35% of doctors are female, representing a 7% improvement from 28% in 2018. Additionally, the upper-income/high-income countries had more female doctors (52%) than the rest of the income group levels, a proportion higher than the regional average (35%). Despite the pharmacists having marginally more male workers (59%) in the region, female pharmacists remained higher in upper-income/high-income countries (58%) compared with the rest of the economies (see figure 7a). Also, low-income countries recorded a higher proportion of male nursing personnel (43%) than the rest of the income groups.

#### Distribution by age

Generally, 82% of the workforce in the region is below 45 years old. Nurses have the highest proportion (89%) of those below the age of 45, followed by pharmacists (88%), midwives (85%), medical doctors (80%), community health workers (76%) and dentists (72%) (see figure 7b). Less than 10% of the workforce are more than 55 years old across the selected occupations, except for nurses, dentists and community health workers in high-income/upper-middle-income countries and medical doctors in lower-middle-income economies.

### Training and education capacity

Based on data from 45 out of 47 countries (excluding Mali and São Tomé and Príncipe), the health professions education capacity in the WHO African Region has improved, with annual education output of health workers increasing from 148 357 graduates in 2018 to well over 255 000 in 2022, representing more than 70% growth. By occupation group, 59% of the training outputs are nursing and midwifery personnel (46% and 13%, respectively), 12% medical doctors and 29% representing other health workers. Combined, doctors, nurses and midwives make up about 71% of the health workers trained in the African region.

**Figure 8:**
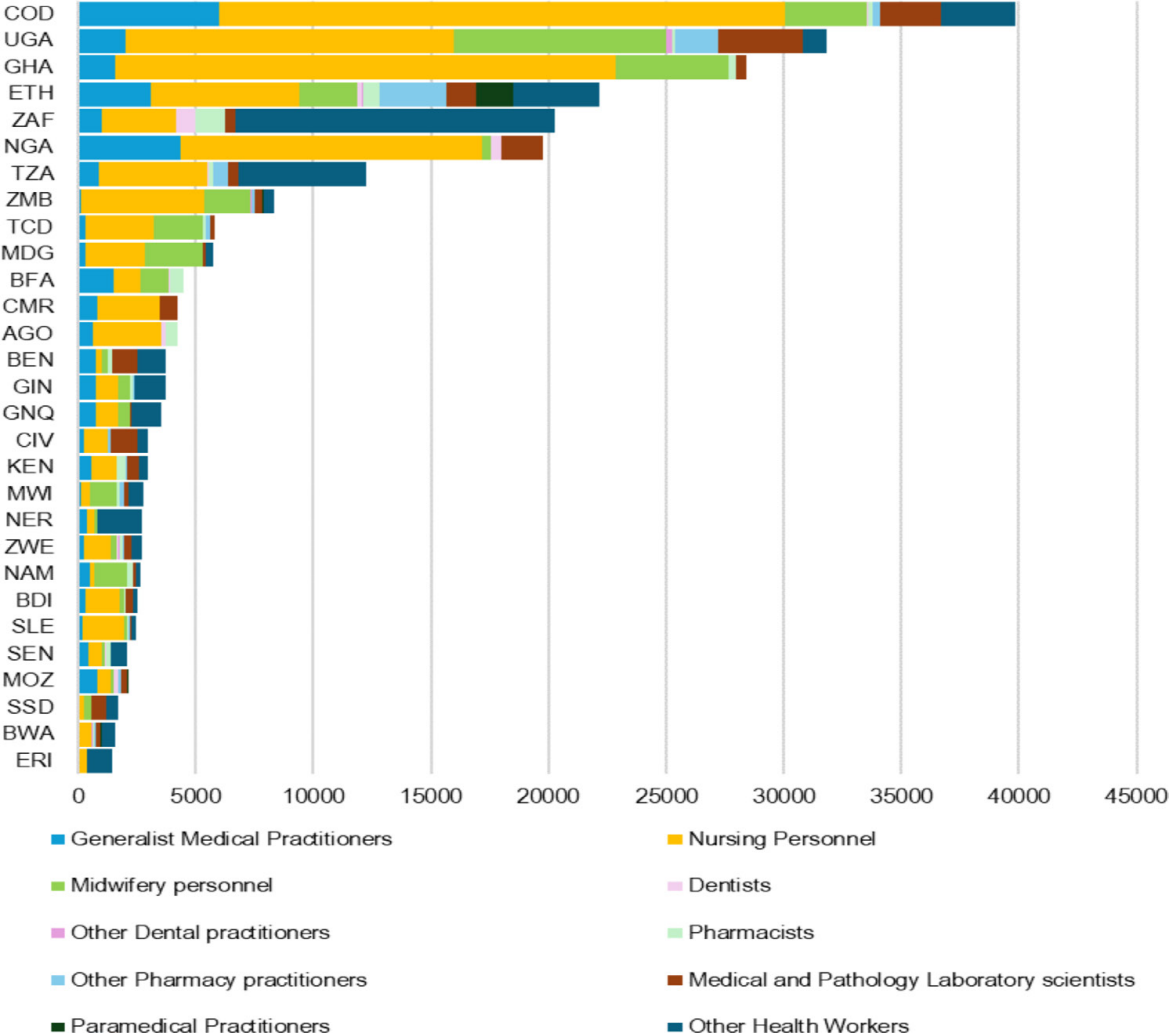
Annual health workforce training and education outputs.

Six countries (Burkina Faso, DRC, Ethiopia, Ghana, Nigeria and Uganda) produce more than 1000 medical graduates annually in the African region. Likewise, six countries (DRC, Ethiopia, Ghana, Nigeria, Uganda and Zambia) produce more than 5000 nurses annually (figure 8). However, for some of these countries, their production level is inadequate relative to their population’s health needs.

Health worker training output is at a ratio of 1:10 when compared with the stock of health workers in the African region. Thus, across all countries, the health worker education annual output is about 10% of the existing stock (replenishment rate). Typically, for every medical doctor trained, three nurses are trained; however, there is a wide variation in the ratio. For example, four countries (Ghana, Lesotho, Rwanda and Zambia) are training more than 10 nurses for every medical doctor trained.

**Figure 9:**
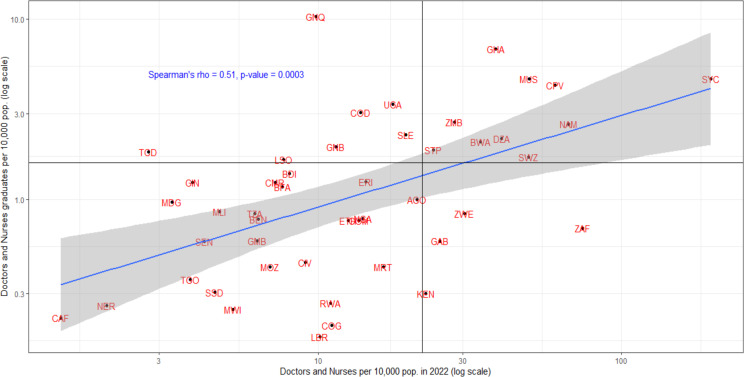
Association between graduates and the density of doctors and nurses.

However, 15 out of 42 countries (36%) with comparable data are producing doctors and nurses faster than their absorption rate into the practising stock. Thus, some of those trained are either not finding jobs or are not included in the practising stock, or are finding job opportunities outside the health sector and/or abroad. On the other hand, 14 out of 42 countries (33%) with comparable data have their domestic training output slower than a relatively fast expansion of the stock (figure 9). Thus, some countries may be relying on foreign training and recruitment to complement the local pipeline of health workforce training output.

### Health workforce unemployment

Systematic health workforce unemployment data from many countries are unavailable in publicly accessible forms. To arrive at an approximate summary metric, data on the unemployment of health workers were extracted from HLMA reports for 10 countries that used a similar methodology for assessment. From the subset of 10 countries, the crude rate of health worker unemployment was estimated to be 26.57% (95% CI 14.03%, 39.11%). Even after standardisation, the unemployment rate was roughly 24.21% (95% CI 15.32%, 33.10%) (table 5). This should, however, be interpreted with caution as the data were collected for different years across these countries and may not necessarily be from comparable sources.

**Table 5:** Health workforce unemployment in selected countries in Africa

S/N	Country	Year	Total unemployed health workers	Proportion of unemployed (%) (HLMA)	Active stock of all health workers (SDG 3c occupations)	Total health workforce (active stock+ unemployed)	Estimated unemployment rate (%) (standardised)	Source
1	Ghana	2023	118 488	39.71	160 787	279 275	42.43	HLMA report (2023)
2	Uganda	2023	75 577	47.55	121 326	196 903	38.38	HLMA report (2023)
3	Zambia	2023	46 713	56.57	69 982	116 695	40.03	Draft HLMA report (2023)
4	South Africa	2019	45 000	11.08	468 294	513 294	8.77	HRH strategy paper (2020)
5	Kenya	2021	27 243	14.34	132 496	159 739	17.05	HLMA report (2023)
6	Mozambique	2023	10 622	17.53	40 757	51 379	20.67	Draft HLMA report (2023)
7	Sierra Leone	2019	4899	33.86	20 253	25 152	19.48	HLMA report (2019)
8	Rwanda	2019	2891	13.41	18 462	21 353	13.54	HLMA report (2019)
9	Lesotho	2021	1096	7.16	5302	6398	17.13	HLMA report (2021)
10	Eswatini	2023	907	12.67	6208	7115	12.75	HLMA report (2023)
	Overall			26.57 (95% CI 14.03%, 39.11%)			24.21% (95% CI 15.32%, 33.01%)	

HLMA, health labour market analysis; HRH, human resources for health; SDG, Sustainable Development Goals.

## Discussion

The comprehensiveness of the findings presented here can be attributed to the improvement in health workforce data availability and quality as more countries have implemented the NHWA. Our findings confirm other global experiences^18^ that when countries fully implement the NHWA, the availability and quality of the data significantly improve.^19^

The analysis showed an improvement in the African region’s health workforce situation over the last decade. The stock of health workers in 2022 increased almost threefold from the 2013 figure, and the density more than doubled between 2013 and 2022. This has been attributed to the increasing investment in the capacity of health professions education institutions to produce more health workers in the African region, improved data availability and the resilience of health professions education institutions in sustaining production amidst disruptive emergencies, such as the COVID-19 pandemic.

In a positive light, about 72% of the health workforce are female, which is in the same order of magnitude as the 70% global average.^20^ The medical profession has progressively included more women, with the proportion of female doctors increasing from 28% in 2018 to 35% in 2022. Despite the majority of health workers being female, their representation at leadership level has been estimated to be only one in four,^20 21^ and they face a wide pay gap compared with their male counterparts. It is time to act—women must be supported and encouraged to contribute to their full potential and be paid equally for the value of their work.

Despite the overall improvement in stock and density of health workers in the region, the density of health workers in the Central African Republic, Chad, Eritrea, Gabon, The Gambia, Guinea, Lesotho, Liberia, Madagascar and Mauritius reduced between 2018 and 2022, a phenomenon that is attributed to the slow growth in training output, inability to absorb new graduates rapidly, emigration and increasing population. Additionally, although the stock and density of health workers in the region have improved, the number is inadequate to meet the health needs projected in various studies.^22^,^26^

This study also found that the number of health workers produced in the region has increased by 70% between 2018 and 2022. However, the training outputs of health workers reported in this study do not include community health workers due to the wide variations in education modalities and recognition within countries. Thus, standardising and institutionalising community health worker education based on prototype competency-based curricula are critical to professionalising the occupation and mainstreaming the reporting of data on the occupation through the NHWA. Also, health workforce analytics such as HLMA and development of national health workforce strategies should seamlessly include community health workers.^27^

Overall, the modest success in the health workforce development in countries within the WHO African Region is notably due to increased HLMA, evidence-based health workforce planning and policy making, and advocacy at the regional, subregional and national levels, including the development of the Africa Health Workforce Investment Charter.^28^ These initiatives must be encouraged and supported to ensure universal access to qualified, skilled and motivated health workers to achieve UHC in the region.

### Limitations

Countries did not report data on some occupations in 2022; hence, the latest available data were used. For others, inconsistent data were reported compared with previously reported data from the same countries through the NHWA. Data triangulation was done using HLMA reports, health workforce strategies and other publicly available government data sources to address this limitation. Additionally, low reporting rates for some occupations, especially non-clinical occupations, affected data completeness. Future efforts on NHWA implementation should target occupations that are not routinely reported by countries to strengthen the availability of comparable country data.

## Conclusion

This study provides new insights that can help shape policy and investment dialogues to strengthen the health workforce across the region towards UHC and ensuring health security. It highlights significant improvements since the last decade and also points out emerging areas of priorities for action. The upward trajectories of the stock, density and educational output are commendable. Nevertheless, countries with special challenges leading to decreasing stock and density will require indepth HLMAs to diagnose and provide plausible solutions to offset the negative trajectory. Continuous technical and financial support for member countries is essential in developing the required number and skill mix of health workers towards the progressive realisation of the UHC aspirations in the African region.

## Supplementary material

10.1136/bmjgh-2024-015952 online supplemental file 1

10.1136/bmjgh-2024-015952 online supplemental file 2

## Data Availability

All data relevant to the study are included in the article or uploaded as supplementary information.
