# Supplementary material for: State of the health workforce in the WHO African Region: decade review of progress and opportunities for policy reforms and investments
Source: BMJ Glob Health. 2024 Nov 25;7(Suppl 1):e015952. doi: 10.1136/bmjgh-2024-015952 (PMC11733074; doi:10.1136/bmjgh-2024-015952)

Supplementary material 1: Trends in the stock and densities of selected occupations

Medical Doctors, Nurses and Midwifery personnel (Combined):There were 2.4 million doctors, nurses and midwives in 2022 which was an increase from 1.8 million in 2018 and about 800,000 in 2013 in the African region. Further, there were 25 doctors, nurses and midwives in 2022 per 10,000 population, compared to 21 in 2018 and 11 in 2013. Thirteen (13) countries (Uganda, São Tomé and Príncipe, Gabon, Zambia, Botswana, Zimbabwe, Ghana, Eswatini, Mauritius, Cabo Verde, Namibia, South Africa, and Seychelles) had already achieved the MDG target with seven of them (Ghana, Eswatini, Mauritius, Cabo Verde, Namibia, South Africa, and Seychelles) having achieved the SDG target for these occupations of 44.5 per 10,000.

Medical doctors: The aggregate medical doctors' stock in the region grew from 115,272 in 2013 to 369,145 in 2022 representing an annual rate of 13.8%. The increase was not at the same pace between 2018 and 2022 as compared to the previous five years. The possible contributing factors can include increasing emigration, impact of COVID-19 (deaths, long COVID-19, delayed graduation, social-economic), poor quality of data. Despite improved capacity to train health workers, the overall stock of medical doctors decreased marginally between 2018 and 2022. The magnitude of decrease rages between 13,000 and 30,000 over the four-year period. This decrease stock was recorded in 28% of the countries (13 out of the 47 countries). The density had a higher annual growth of 15.7%, compared to the stock, with the regional density of 1.44 in 2013 increasing to 5.33 per 10,000 in 2022. The top ten most improved countries in their density include: Seychelles, Cabo Verde, Algeria, South Africa, Namibia, Eswatini, Gabon, Zambia, Comoros, and Angola. Least improvement in density were predominantly in Central African Republic, Côte d'Ivoire, Mauritius, Niger, and The Gambia, (see Supplementary Figure 1).

Nurses and midwives Personnel: The nursing personnel stock in the African Region increased from 479,279 in 2013 to 1,698,828 in 2022 (nurses and their associate professionals) which represents an annual rate of 15.1%. However, in nine countries the stock in 2022 decreased compared to 2018. These counties are Central African Republic, Chad, Gabon, Gambia, Ghana, Guinea, Lesotho, Liberia, and Mauritius. The possible contributing factors can include increasing emigration, impact of COVID-19 (deaths, long COVID-19, delayed graduation, social-economic), potentially data quality issues ((see Supplementary Figure 1). On the other hand, the density grew by 8.8% annually from 7.89 in 2013 to 16.81 in 2022. Top ten most improved countries in nurses' density include: Seychelles, South Africa, Namibia, Zambia, Algeria, Ghana, Gabon, Angola, Zimbabwe, and Kenya with the five least improved over the ten-year period being Cameroon, Chad, Gambia, Guinea, and São Tomé and Príncipe.

Since some countries reported their nurses together with the midwifery personnel stock, the combined stock and density of nurses and midwives was also computed. It was found that increase to 2,033,359 in 2022 from 652,548 in 2013 which is as a results of an annual growth rate of 13.5%. The top ten

countries with the highest increase in their nurses and midwives' stock were: Algeria, Angola, DRC, Ethiopia, Ghana, Kenya, Nigeria, South Africa, Uganda, Zambia and Zimbabwe. Over the same period, the density per 10,000 increased from 9.12 in 2013 to 19.37 in 2022. The highest increases in the density were in Angola, Gabon, Ghana, Lesotho, Namibia, Seychelles, South Africa, Zambia, and Zimbabwe and the countries with the least changes were Botswana, Cameroon, Gambia, Guinea, and São Tomé and Príncipe (see Supplementary Figure 1).

Dentists: The dentists' stock was 34,405 in 2022 which was an increase from 9,015 in 2013. This represented an annual rate of 16% over the ten-year period. The 2022 stock was a decline from 37,007 in 2018. This decline was witness in 36% (17 out of 47) of the countries and could be attributable to COVID-19 effects. However, after boosting this stock by the Dental Assistants and Therapists gives 56,772 in 2022 from 14,817 in 2013. On the other hand, the density increased at an annual rate of 10.3% to stand at 0.37 per 10,000 in 2022 from 0.15 in 2013. The top ten countries with the most improved densities were Seychelles, Algeria, Cabo Verde, Namibia, Mauritius, Nigeria, Eswatini, Eritrea, and Lesotho, while the least improved (or dropped) were Côte d'Ivoire, Equatorial Guinea, Niger, South Africa, Togo, and Zimbabwe (see Supplementary Figure 1).

Pharmacists: Over the ten-year period, the stock of pharmacists increased at an annual rate of 9.9% to reach 101,401 in 2022 from 43,321 in 2013. However, this high annual growth rate was highly influence by improved reporting between 2018 and 2022, where the annual growth rate 2.7%. Despite the sustained overall growth in the stock, the data shows a decline in the density in 49% (23 out of 47) of the countries between 2018 and 2022. During the same period, their density increased at an annual rate of 11.9% from 0.26 in 2013 to 0.73 in 2022. The top ten countries with the most improved pharmacists' densities in the duration under consideration were Seychelles, Namibia, Ethiopia, Lesotho, Botswana, Ghana, Gabon, São Tomé and Príncipe, Eritrea, and Eswatini with the least or dropped being Benin, Cameroon, Côte d'Ivoire, Gambia, Mauritius, Senegal, and South Africa (see Supplementary Figure 1).

Supplementary Figure 1: The trend of doctors, nurses and midwives' density per 10,000 between 2013 and 2022

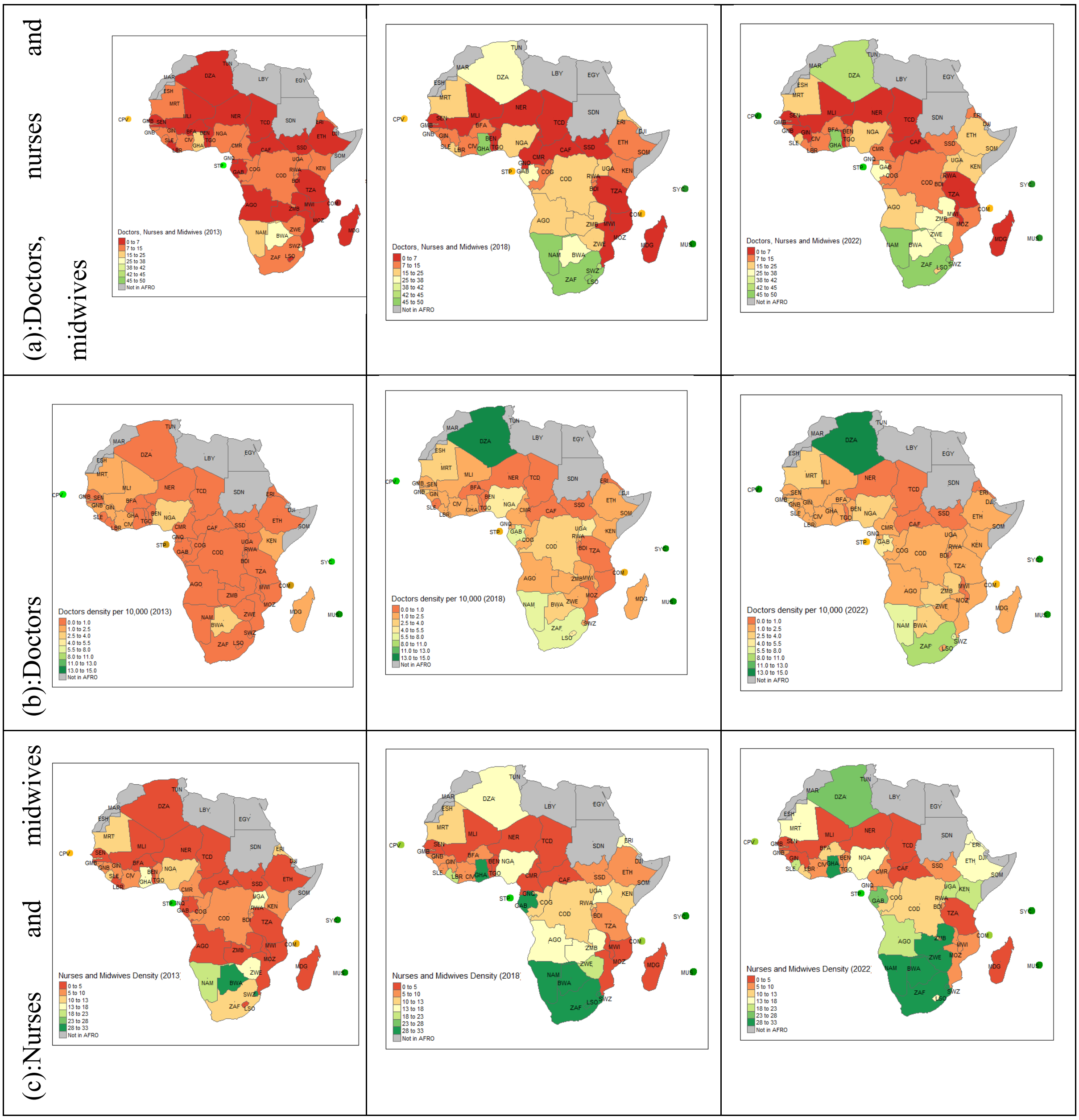

Supplement: online supplemental file 1 [file bmjgh-7-Suppl_1-s001.pdf]
