# Supplementary material for: State of the health workforce in the WHO African Region: decade review of progress and opportunities for policy reforms and investments
Source: BMJ Glob Health. 2024 Nov 25;7(Suppl 1):e015952. doi: 10.1136/bmjgh-2024-015952 (PMC11733074; doi:10.1136/bmjgh-2024-015952)
Supplement: online supplemental file 2 [file bmjgh-7-Suppl_1-s002.pdf]

The state of health workforce in the WHO Africa Region: A decade review of progress and opportunities for policy reforms and investments.

Authors:

James Avoka Asamani[1,4], San Boris Kouadjo Bediakon[1], Mathieu Boniol[2], Joseph K Mung'atu[3], Christmal Dela Christmals[4], Sunny C. Okoroafor[1], Adam Ahmat[1], Maritza Titus[1], Jean Benard Moussounda[1], Hilary Kipruto[5], Kasonde Grace Mwinga[6], Joseph Waogodo Cabore[7], Rebecca Matshidiso Moeti[8].

1. Health Workforce Unit, Universal Health Coverage - Life Course, World Health Organization Regional Office for Africa, Brazzaville, Congo
2. Health Workforce Department, World Health Organization Headquarters, Geneva, Switzerland
3. Department of Statistics and Actuarial Sciences, Jomo Kenyatta University of Agriculture and Technology, Juja, Kenya.
4. Centre for Health Professions Education, Faculty of Health Sciences, North-West University, Potchefstroom, South Africa.
5. Health Information Systems Unit, Universal Health Coverage - Life Course, World Health Organization Regional Office for Africa, Brazzaville, Congo.
6. Office of the Director, Universal Health Coverage - Life Course, World Health Organization Regional Office for Africa, Brazzaville, Congo.
7. Office of the Director, Programme Management, World Health Organization Regional Office for Africa, Brazzaville, Congo.
8. Office of the Regional Director, World Health Organization Regional Office for Africa, Brazzaville, Congo.

Supplementary materials – Data Tables

Appendix Table 1: Data availability for the countries across the 33 occupations for 2013, 2018 and 2022 4
Appendix Table 2: *The trends of SDG 3c occupations versus other workers stock in WHO African Region between 2013 and 2022*5
Appendix Table 3: *The trends of SDG 3c occupations versus other workers' density in WHO AFRICAN REGION between 2013 and 2022* 7
Appendix Table 4: The trends of medical doctors' stock and density in WHO AFRICAN REGION between 2013 and 2022 9
Appendix Table 5: The trends of nursing personnels' stock and density in WHO AFRICAN REGION between 2013 and 2022 10
Appendix Table 6: The trends of midwifery personnels' stock in WHO AFRICAN REGION between 2013 and 2022 11
Appendix Table 7: The trends of nurses and midwives' stock and density in WHO AFRICAN REGION between 2013 and 2022 12
Appendix Table 8: The trends of dentists and dental assistants & therapists' stock and density in WHO AFRICAN REGION between 2013 and 2022 13
Appendix Table 9: The trends of Pharmacists and Pharmaceutical Technicians and Assistants' stock and density in WHO AFRICAN REGION between 2013 and 2022 15
Appendix Table 10: The trends of community health workers' stock and density in WHO AFRICAN REGION between 2013 and 2022 17
Appendix Table 11: The trends of dieticians and nutritionists' stock and density in WHO AFRICAN REGION between 2013 and 2022 18
Appendix Table 12: The trends of Environmental and occupational health & hygiene workers' stock and density in WHO AFRICAN REGION between 2013 and 2022 19
Appendix Table 13: The trends of managerial staff stock and density in WHO AFRICAN REGION between 2013 and 2022 20
Appendix Table 14: The trends of Medical and dental Prosthetic Technicians' stock and density in WHO AFRICAN REGION between 2013 and 2022 21
Appendix Table 15: The trends of Medical and Pathology Laboratory scientists' stock and density in WHO AFRICAN REGION between 2013 and 2022 22
Appendix Table 16: The trends of Medical and Pathology Laboratory Technicians' stock and density in WHO AFRICAN REGION between 2013 and 2022 23
Appendix Table 17: The trends of Medical Imaging and Therapeutic Equipment Technicians' stock and density in WHO AFRICAN REGION between 2013 and 2022 24
Appendix Table 18: The trends of Medical Records and Health Information Technicians' stock and density in WHO AFRICAN REGION between 2013 and 2022 25
Appendix Table 19: The trends of Optometrists and Ophthalmic Opticians' stock and density in WHO AFRICAN REGION between 2013 and 2022 26
Appendix Table 20: The trends of Other non-medical professional staff stock and density in WHO AFRICAN REGION between 2013 and 2022 27
Appendix Table 21: The trends of Other non-medical support staff stock and density in WHO AFRICAN REGION between 2013 and 2022 28
Appendix Table 22: The trends of Paramedical Practitioners' stock and density in WHO AFRICAN REGION between 2013 and 2022 29
Appendix Table 23: The trends of Physiotherapists and physiotherapy assistants' stock and density in WHO AFRICAN REGION between 2013 and 2022 30

Appendix Table 24: The trends of SDG 3c occupations versus other workers stock in WHO AFRICAN REGION between 2013 and 2022 31
Appendix Table 25: The trends of SDG 3c occupations versus other workers' density in WHO AFRICAN REGION between 2013 and 2022 33
Appendix Table 26: The stock and density of doctors, nurses and midwives between 2013 and 2022 35

Appendix Table 1: Data availability for the countries across the 33 occupations for 2013, 2018 and 2022

No	Country	Number of occupations reported in 2013	Number of occupations reported in 2018	Number of occupations reported in 2022
**1**	Algeria	5	30	30
2	Angola	4	28	32
3	Benin	9	25	26
4	Botswana	8	25	25
5	Burkina Faso	13	30	32
6	Burundi	11	25	27
7	Cameroon	7	16	33
8	Cabo Verde	4	29	29
9	Central African Republic	10	26	27
10	Chad	6	28	30
11	Comoros	8	17	17
12	Congo	8	20	21
13	Cote d'Ivoire	17	28	29
14	DRC	11	21	21
15	Equatorial Guinea	2	13	19
16	Eritrea	4	20	23
17	Eswatini	5	29	32
18	Ethiopia	4	27	28
19	Gabon	4	33	33
20	Gambia	16	23	25
21	Ghana	12	28	31
22	Guinea	8	19	20
23	Guinea-Bissau	8	29	29
24	Kenya	9	18	25
25	Lesotho	2	29	29
26	Liberia	4	24	24
27	Madagascar	9	25	25
28	Malawi	6	23	29
29	Mali	5	23	23
30	Mauritania	6	26	26
31	Mauritius	4	26	30
32	Mozambique	17	28	31
33	Namibia	4	28	31
34	Niger	8	24	26
35	Nigeria	7	26	26
36	Rwanda	13	18	20
37	Sao Tome and Principe	6	14	14
38	Senegal	15	31	32
39	Seychelles	4	20	24
40	Sierra Leone	8	25	26
41	South Africa	12	25	28
42	South Sudan		27	27
43	Tanzania	16	26	27
44	Togo	11	32	32
45	Uganda	16	23	24
46	Zambia	12	24	27
47	Zimbabwe	20	30	33

Appendix Table 2: *The trends of SDG 3c occupations versus other workers stock in WHO African Region between 2013 and 2022*

S/No	Country	SDG 3c occupations workforce* 2013	2018	2022	Other workers 2013	2018	2022	Total 2013	2018	2022
1	Algeria	11,139	175,647	220,805	200	240,105	366,964	11,339	415,752	587,769
2	Angola	30	60,468	78,870	602	50,395	58,067	632	110,863	136,937
3	Benin	7,923	5,769	11,051	15,066	11,707	13,973	22,989	17,476	25,024
4	Botswana	8,191	9,284	9,739	1,000	7,514	8,210	9,191	16,798	17,949
5	Burkina Faso	8,983	19,646	27,591	4,585	7,601	10,576	13,568	27,247	38,168
6	Burundi	7,170	10,533	10,709	44	37,621	42,120	7,214	48,154	52,830
7	Cameroon	22,321	14,532	23,282	-	16,843	8,354	22,321	31,375	31,636
8	Cape-Verde	711	1,666	3,734	7,662	5,297	2,476	8,373	6,963	6,210
9	Central African Republic	1,180	2,027	1,344	384	1,029	4,354	1,564	3,056	5,698
10	Chad	4,702	6,992	6,339	1,664	3,800	33,924	6,366	10,792	40,262
11	Comoros	452	1,541	1,868	841	1,215	754	1,293	2,756	2,622
12	Congo	4,571	6,962	7,792	4,923	3,307	4,346	9,494	10,269	12,138
13	Cote d'Ivoire	23,221	29,443	35,290	5,120	51,343	37,741	28,341	80,786	73,031
14	DRC	68,069	141,290	141,402	3,066	85,872	15,905	71,135	227,162	157,308
15	Equatorial Guinea	-	662	1,781	6	1,994	2,902	6	2,656	4,683
16	Eritrea	4,434	6,397	6,180	-	5,483	7,859	4,434	11,880	14,039
17	Eswatini	3,735	5,570	6,208	4,661	12,031	10,642	8,396	17,601	16,850
18	Ethiopia	6,925	108,826	229,225	511	160,072	188,294	7,436	268,898	417,519
19	Gabon	437	8,562	7,468	1,295	4,369	6,195	1,732	12,931	13,663
20	Gambia	2,979	2,284	2,205	1,007	2,625	7,047	3,986	4,909	9,253
21	Ghana	51,421	143,265	160,787	24,391	62,817	77,240	75,812	206,082	238,027
22	Guinea	8,275	11,176	9,201	6,700	18,091	18,091	14,975	29,267	27,292
23	Guinea-Bissau	1,234	2,097	2,712	496	6,168	6,479	1,730	8,265	9,191
24	Kenya	52,221	72,002	132,496	13,628	92,554	153,550	65,849	164,556	286,046
25	Lesotho	-	11,621	5,302	216	25,051	23,691	216	36,672	28,993
26	Liberia	974	11,203	7,129	2,036	13,864	23,098	3,010	25,067	30,227
27	Madagascar	10,489	14,669	14,098	174	44,152	50,968	10,663	58,821	65,067
28	Malawi	3,963	11,568	12,418	-	14,833	26,224	3,963	26,401	38,642
29	Mali	8,189	12,833	14,537	-	14,277	23,767	8,189	27,110	38,304
30	Mauritania	4,836	6,798	9,375	244	11,463	11,463	5,080	18,261	20,838
31	Mauritius	6,820	9,444	7,847	-	6,377	7,398	6,820	15,821	15,245
32	Mozambique	14,014	19,598	40,757	5,346	76,726	139,367	19,360	96,324	180,124
33	Namibia	4,178	15,602	18,611	-	10,043	8,448	4,178	25,645	27,059
34	Niger	5,477	6,168	6,177	6,560	3,596	6,818	12,037	9,764	12,995
35	Nigeria	261,989	441,434	495,916	331,077	537,756	303,917	593,066	979,190	799,833

S/No	Country	SDG 3c occupations workforce* 2013	2018	2022	Other workers 2013	2018	2022	Total 2013	2018	2022
**36**	Rwanda	11,592	17,779	18,462	2,695	49,680	63,680	14,287	67,459	82,142
**37**	Sao Tome and Principe	536	559	613	28	1,105	828	564	1,664	1,441
**38**	Senegal	6,196	10,926	11,285	4,932	34,349	29,940	11,128	45,275	41,225
**39**	Seychelles	530	2,020	2,592	-	525	594	530	2,545	3,187
**40**	Sierra Leone	8,293	7,320	20,253	4,557	2,288	18,899	12,850	9,608	39,151
**41**	South Africa	90,079	354,250	468,294	23,469	214,207	191,863	113,548	568,457	660,157
**42**	South Sudan	-	6,413	8,617	-	10,147	12,908	-	16,560	21,525
**43**	Tanzania	23,402	39,366	49,815	15,093	69,773	138,070	38,495	109,139	187,885
**44**	Togo	2,532	4,137	5,575	638	22,503	21,202	3,170	26,640	26,777
**45**	Uganda	51,995	77,940	121,326	207,921	190,898	215,144	259,916	268,838	336,470
**46**	Zambia	4,278	30,782	69,982	5,507	32,379	42,024	9,785	63,161	112,006
**47**	Zimbabwe	21,148	35,628	62,258	15,850	36,886	42,581	36,998	72,514	104,840
**48**	**WHO AFRICAN REGION**	**841,834**	**1,994,699**	**2,609,321**	**724,195**	**2,312,731**	**2,488,958**	**1,566,029**	**4,307,430**	**5,098,278**

SDG 3c occupations* - Doctors, Nurses, midwives, pharmacists and Dentists

Appendix Table 3: *The trends of SDG 3c occupations versus other workers' density in WHO AFRICAN REGION between 2013 and 2022*

SNo	Country	SDG 3c occupations workforce* Density per 10,000(2013)	Density per 10,000(2018)	Density per 10,000(2022)	Other workers Density per 10,000(2013)	Density per 10,000(2018)	Density per 10,000(2022)	Total Density per 10,000(2013)	Density per 10,000(2018)	Density per 10,000(2022)
1	Algeria	2.93	41.89	49.17	0.05	57.07	81.72	2.98	98.96	130.90
2	Angola	0.01	19.34	22.16	0.23	16.09	16.30	0.24	35.43	38.46
3	Benin	7.69	4.83	8.28	14.61	9.79	10.45	22.30	14.62	18.73
4	Botswana	36.94	37.87	37.03	4.51	30.54	31.09	41.45	68.41	68.12
5	Burkina Faso	5.09	9.63	12.17	2.60	3.73	4.66	7.69	13.36	16.83
6	Burundi	7.06	9.16	8.31	0.04	32.73	32.68	7.11	41.90	40.99
7	Cameroon	10.32	5.80	8.34	-	6.72	2.99	10.32	12.51	11.33
8	Cape-Verde	13.17	29.17	62.96	141.90	92.38	41.40	155.07	121.55	104.36
9	Central African Republic	2.46	3.98	2.41	0.80	2.02	7.80	3.26	6.00	10.21
10	Chad	3.56	4.48	3.58	1.26	2.44	19.13	4.82	6.92	22.71
11	Comoros	6.46	19.85	22.33	12.02	15.65	9.01	18.49	35.50	31.33
12	Congo	9.47	12.80	13.05	10.20	6.08	7.28	19.66	18.87	20.33
13	Cote d'Ivoire	10.33	11.55	12.53	2.19	20.12	13.39	12.53	31.67	25.92
14	DRC	9.27	16.22	14.28	0.42	9.86	1.61	9.68	26.08	15.89
15	Equatorial Guinea	-	4.41	10.63	0.05	13.24	17.30	0.05	17.65	27.93
16	Eritrea	13.45	18.57	16.77	-	15.91	21.33	13.45	34.48	38.11
17	Eswatini	33.40	48.00	51.66	41.68	103.65	88.54	75.08	151.65	140.20
18	Ethiopia	0.71	9.79	18.58	0.05	14.40	15.25	0.77	24.19	33.83
19	Gabon	2.30	39.06	31.26	6.81	19.72	25.85	9.11	58.78	57.11
20	Gambia	14.02	9.34	8.15	4.72	10.71	26.02	18.74	20.05	34.17
21	Ghana	18.68	46.41	48.03	8.86	20.29	22.61	27.54	66.70	70.64
22	Guinea	7.49	8.90	6.64	6.06	14.41	13.05	13.55	23.31	19.69
23	Guinea-Bissau	7.27	10.89	12.88	2.92	32.02	30.76	10.19	42.91	43.64
24	Kenya	11.66	14.41	24.52	3.04	18.43	28.24	14.70	32.85	52.76
25	Lesotho	-	52.87	22.99	1.04	113.93	102.69	1.04	166.80	125.68
26	Liberia	2.20	22.91	13.44	4.60	28.36	43.56	6.80	51.27	57.00
27	Madagascar	4.45	5.46	4.76	0.07	16.43	17.21	4.52	21.89	21.97
28	Malawi	2.47	6.30	6.09	-	8.08	12.85	2.47	14.37	18.93
29	Mali	4.82	6.44	6.43	-	7.15	10.51	4.82	13.59	16.95
30	Mauritania	12.92	15.92	19.79	0.65	26.82	24.18	13.57	42.74	43.98
31	Mauritius	52.84	72.91	60.38	-	49.14	56.82	52.84	122.04	117.20
32	Mozambique	5.55	6.66	12.36	2.10	25.97	42.25	7.65	32.63	54.61
33	Namibia	18.95	64.85	72.50	-	39.79	32.85	18.95	104.65	105.35
34	Niger	2.94	2.73	2.36	3.52	1.59	2.60	6.45	4.32	4.96
35	Nigeria	14.99	22.25	22.69	18.95	26.80	13.89	33.94	49.05	36.58

SNo	Country	SDG 3c occupations workforce* Density per 10,000(2013)	Density per 10,000(2018)	Density per 10,000(2022)	Other workers Density per 10,000(2013)	Density per 10,000(2018)	Density per 10,000(2022)	Total Density per 10,000(2013)	Density per 10,000(2018)	Density per 10,000(2022)
**36**	Rwanda	10.44	14.19	13.40	2.41	39.64	46.22	12.85	53.83	59.62
**37**	Sao Tome and Principe	27.66	26.45	26.97	1.45	52.28	36.42	29.11	78.73	63.39
**38**	Senegal	4.56	7.02	6.52	3.63	21.99	17.28	8.19	29.00	23.80
**39**	Seychelles	54.80	195.92	242.01	-	50.73	54.45	54.80	246.65	296.47
**40**	SierraLeone	11.91	9.31	23.53	6.54	2.75	21.94	18.45	12.06	45.48
**41**	South Africa	16.72	61.78	78.19	4.36	36.78	31.95	21.08	98.56	110.14
**42**	South Sudan	-	6.17	7.90	-	9.74	11.81	-	15.91	19.70
**43**	Tanzania	4.75	6.78	7.61	3.05	12.00	21.08	7.80	18.77	28.68
**44**	Togo	3.56	5.14	6.30	0.90	27.90	23.93	4.46	33.04	30.24
**45**	Uganda	14.74	18.77	25.68	58.88	45.98	45.26	73.62	64.75	70.94
**46**	Zambia	2.81	17.26	34.96	3.58	18.15	20.98	6.39	35.41	55.94
**47**	Zimbabwe	15.60	23.67	38.15	11.67	24.48	25.90	27.27	48.15	64.04
**48**	**WHO AFRICAN REGION**	**11.14**	**23.58**	**26.82**	**8.35**	**26.69**	**27.34**	**19.49**	**50.27**	**54.17**

Appendix Table 4: The trends of medical doctors' stock and density in WHO AFRICAN REGION between 2013 and 2022

		Medical Doctors* stock			Medical Doctors* density		
S No	Country	2013	2018	2022	Density per 10,000 (2013)	Density per 10,000 (2018)	Density per 10,000 (2022)
1	Algeria	-	73,168	75,512	-	17.45	16.82
2	Angola	-	6,593	8,693	-	2.11	2.44
3	Benin	-	908	2,608	-	0.76	1.95
4	Botswana	819	925	992	3.69	3.77	3.77
5	Burkina Faso	803	1,684	3,323	0.46	0.83	1.47
6	Burundi	554	1,084	1,015	0.55	0.94	0.79
7	Cameroon	1,842	3,259	3,759	0.85	1.30	1.35
8	Cape-Verde	308	452	2,644	5.70	7.91	44.58
9	Central African Republic	324	335	146	0.67	0.66	0.26
10	Chad	573	803	1,507	0.43	0.51	0.85
11	Comoros	123	220	354	1.76	2.83	4.23
12	Congo	-	544	1,042	-	1.00	1.75
13	Cote d'Ivoire	5,240	5,602	6,328	2.33	2.20	2.25
14	DRC	6,418	31,546	21,290	0.87	3.62	2.15
15	Equatorial Guinea	-	246	275	-	1.64	1.64
16	Eritrea	172	280	299	0.52	0.81	0.81
17	Eswatini	-	278	687	-	2.40	5.72
18	Ethiopia	-	11,263	23,741	-	1.01	1.92
19	Gabon	-	1,368	1,265	-	6.24	5.30
20	Gambia	213	298	274	1.00	1.22	1.01
21	Ghana	2,730	4,406	4,726	0.99	1.43	1.41
22	Guinea	1,844	2,787	3	1.67	2.22	2.12
23	Guinea-Bissau	354	578	531	2.09	3.00	2.52
24	Kenya	8,682	8,042	12,792	1.94	1.61	2.37
25	Lesotho	-	998	537	-	4.54	2.33
26	Liberia	168	693	954	0.38	1.42	1.80
27	Madagascar	3,523	5,230	5,230	1.49	1.95	1.77
28	Malawi	200	2,760	1,104	0.12	1.50	0.54
29	Mali	1,971	2,866	4,402	1.16	1.44	1.95
30	Mauritania	622	1,626	1,273	1.66	3.81	2.69
31	Mauritius	2,046	2,395	1,834	15.85	18.49	14.11
32	Mozambique	1,452	2,473	5,719	0.58	0.84	1.73
33	Namibia	-	1,445	1,597	-	6.01	6.22
34	Niger	946	1,065	706	0.51	0.47	0.27
35	Nigeria	65,759	83,907	86,181	3.76	4.23	3.94
36	Rwanda	1,104	1,648	1,648	0.99	1.32	1.20
37	Sao Tome and Principe	63	60	105	3.25	2.84	4.62
38	Senegal	-	2,883	2,561	-	1.85	1.48
39	Seychelles	93	240	828	9.62	23.28	77.30
40	Sierra Leone	-	566	1,106	-	0.72	1.29
41	South Africa	-	43,503	48,021	-	7.59	8.02
42	South Sudan	-	411	456	-	0.40	0.42
43	Tanzania	1,481	2,885	8,693	0.30	0.50	1.33
44	Togo	361	611	730	0.51	0.76	0.82
45	Uganda	3,370	17,186	9,052	0.96	4.14	1.92
46	Zambia	-	4,021	6,531	-	2.25	3.26
47	Zimbabwe	1,114	3,026	3,132	0.82	2.01	1.92
48	**AFRO**	**115,272**	**339,167**	**366,203**	**1.44**	**3.40**	**5.33**

Medical Doctors* - Generalists, Specialists and the undefined

- Indicates NO DATA

Appendix Table 5: The trends of nursing personnels' stock and density in WHO AFRICAN REGION between 2013 and 2022

		Nursing Personnel*					
S No	**Country**	**2013**	**2018**	**2022**	**Density per 10,000 (2013)**	**Density per 10,000 (2018)**	**Density per 10,000 (2022)**
1	Algeria	-	57,705	104,776	-	13.76	23.33
2	Angola	-	46,082	66,717	-	14.74	18.75
3	Benin	4,880	3,410	5,893	4.73	2.86	4.41
4	Botswana	5,816	7,645	8,028	26.23	31.19	30.52
5	Burkina Faso	6,196	10,325	13,961	3.51	5.06	6.16
6	Burundi	6,573	9,156	9,372	6.48	7.97	7.27
7	Cameroon	18,954	9,925	16,473	8.76	3.96	5.90
8	Cape-Verde	403	768	941	7.46	13.45	15.86
9	Central African Republic	322	1,195	648	0.67	2.35	1.16
10	Chad	3,606	4,799	3,387	2.73	3.08	1.91
11	Comoros	-	747	785	-	9.62	9.38
12	Congo	3,289	5,081	5,573	6.81	9.34	9.33
13	Cote d'Ivoire	8,780	15,163	19,225	3.91	5.95	6.83
14	DRC	59,813	103,785	115,316	8.14	11.92	11.65
15	Equatorial Guinea	-	355	1,375	-	2.36	8.21
16	Eritrea	4,262	4,971	5,007	12.93	14.43	13.59
17	Eswatini	3,735	4,706	5,242	33.40	40.55	43.62
18	Ethiopia	-	61,772	131,262	-	5.56	10.64
19	Gabon	-	6,085	4,752	-	27.76	19.89
20	Gambia	2,386	1,498	1,429	11.23	6.13	5.28
21	Ghana	37,404	125,024	124,204	13.59	40.50	37.10
22	Guinea	5,789	7,195	5,019	5.24	5.73	3.62
23	Guinea-Bissau	727	1,264	1,879	4.28	6.57	8.92
24	Kenya	40,292	59,901	109,659	9.00	11.99	20.30
25	Lesotho	-	6,866	1,235	-	31.24	5.36
26	Liberia	-	8,488	4,424	-	17.36	8.34
27	Madagascar	3,438	4,560	4,560	1.46	1.70	1.54
28	Malawi	3,545	7,957	9,592	2.21	4.33	4.70
29	Mali	4,257	5,225	6,327	2.50	2.62	2.80
30	Mauritania	3,846	4,066	6,503	10.28	9.52	13.73
31	Mauritius	3,963	4,986	4,599	30.70	38.49	35.39
32	Mozambique	6,432	8,153	17,168	2.55	2.77	5.21
33	Namibia	4,110	12,956	15,558	18.64	53.86	60.61
34	Niger	3,533	4,107	4,565	1.89	1.82	1.74
35	Nigeria	74,920	180,709	220,681	4.29	9.11	10.10
36	Rwanda	9,625	13,345	13,501	8.67	10.65	9.80
37	Sao Tome and Principe	442	406	440	22.81	19.21	19.35
38	Senegal	1,682	4,577	4,737	1.24	2.94	2.74
39	Seychelles	419	1,272	1,277	43.32	123.37	119.21
40	Sierra Leone	6,106	5,757	15,666	8.77	7.32	18.20
41	South Africa	58,722	287,458	397,261	10.90	50.13	66.33
42	South Sudan	-	3,726	4,530	-	3.58	4.15
43	Tanzania	20,800	31,940	31,940	4.22	5.50	4.88
44	Togo	1,341	1,966	2,630	1.89	2.44	2.97
45	Uganda	40,149	52,907	74,197	11.38	12.74	15.70
46	Zambia	-	20,746	49,975	-	11.63	24.97
47	Zimbabwe	18,722	27,874	46,537	13.81	18.52	28.51
48	**WHO AFRICAN REGION**	**479,279**	**1,248,604**	**1,698,828**	**7.89**	**15.69**	**16.81**

Nursing Personnel* - Nursing Professionals, Nursing Associate Professionals, Nurses not further defined

- Indicates NO DATA

Appendix Table 6: The trends of midwifery personnels' stock in WHO AFRICAN REGION between 2013 and 2022

		Midwifery personnel*					
**S No**	**Country**	**2013**	**2018**	**2022**	**Density per 10,000 (2013)**	**Density per 10,000 (2018)**	**Density per 10,000 (2022)**
1	Algeria	11,139	8,948	9,070	2.93	2.13	2.02
2	Angola	30	1,438	33	0.01	0.46	0.01
3	Benin	943	1,055	2,055	0.91	0.88	1.54
4	Botswana	1,501	-	-	6.77	-	-
5	Burkina Faso	1,591	6,569	9,294	0.90	3.22	4.10
6	Burundi	35	160	179	0.03	0.14	0.14
7	Cameroon	127	690	1,674	0.06	0.28	0.60
8	Cape-Verde	-	27	27	-	0.47	0.46
9	Central African Republic	514	444	537	1.07	0.87	0.96
10	Chad	451	936	904	0.34	0.60	0.51
11	Comoros	295	487	586	4.22	6.27	7.00
12	Congo	1,282	1,069	1,042	2.66	1.96	1.75
13	Cote d'Ivoire	3,851	5,460	7,425	1.71	2.14	2.64
14	DRC	1,555	3,642	3,039	0.21	0.42	0.31
15	Equatorial Guinea	-	51	26	-	0.34	0.16
16	Eritrea	-	45	67	-	0.13	0.18
17	Eswatini	-	14	14	-	0.12	0.12
18	Ethiopia	6,925	16,161	34,541	0.71	1.45	2.80
19	Gabon	437	733	920	2.30	3.34	3.85
20	Gambia	259	320	379	1.22	1.31	1.40
21	Ghana	11,287	10,625	23,882	4.10	3.44	7.13
22	Guinea	500	866	893	0.45	0.69	0.64
23	Guinea-Bissau	153	157	197	0.90	0.82	0.94
24	Kenya	-	-	137	-	-	0.03
25	Lesotho	-	2,779	2,779	-	12.64	12.05
26	Liberia	806	927	989	1.82	1.90	1.86
27	Madagascar	3,400	3,994	3,994	1.44	1.49	1.35
28	Malawi	48	-	616	0.03	-	0.30
29	Mali	1,961	3,235	1,920	1.15	1.62	0.85
30	Mauritania	368	855	1,340	0.98	2.00	2.83
31	Mauritius	-	567	567	-	4.38	4.36
32	Mozambique	4,265	6,021	13,384	1.69	2.05	4.06
33	Namibia	-	5	17	-	0.02	0.07
34	Niger	917	906	825	0.49	0.40	0.31
35	Nigeria	101,275	120,870	146,787	5.80	6.09	6.72
36	Rwanda	622	1,471	1,990	0.56	1.17	1.44
37	Sao Tome and Principe	31	50	25	1.60	2.37	1.11
38	Senegal	3,946	3,015	3,281	2.90	1.94	1.89
39	Seychelles	-	158	179	-	15.32	16.74
40	Sierra Leone	2,187	391	2,271	3.14	0.50	2.64
41	South Africa	-	-	-	-	-	-
42	South Sudan	-	1,712	3,020	-	1.65	2.77
43	Tanzania	-	-	-	-	-	-
44	Togo	795	1,285	1,864	1.12	1.60	2.11
45	Uganda	7,000	7,237	32,957	1.98	1.74	6.98
46	Zambia	2,773	3,432	9,202	1.82	1.92	4.60
47	Zimbabwe	-	854	9,601	-	0.57	5.88
48	**WHO AFRICAN REGION**	**173,269**	**219,661**	**334,530**	**1.24**	**1.98**	**2.56**

- Indicates NO DATA

Appendix Table 7: The trends of nurses and midwives' stock and density in WHO AFRICAN REGION between 2013 and 2022

		Midwives and nurses			**Density per 10,000 (2013)**	**Density per 10,000 (2018)**	**Density per 10,000 (2022)**
S No	**Country**	**2013**	**2018**	**2022**			
1	Algeria	11,139	66,653	113,846	2.93	15.90	25.35
2	Angola	30	47,520	66,750	0.01	15.19	18.76
3	Benin	5,823	4,465	7,948	5.65	3.74	5.95
4	Botswana	7,317	7,645	8,028	33.00	31.19	30.52
5	Burkina Faso	7,787	16,894	23,255	4.42	8.28	10.26
6	Burundi	6,608	9,316	9,551	6.51	8.11	7.41
7	Cameroon	19,081	10,615	18,147	8.82	4.23	6.50
8	Cape-Verde	403	795	968	7.46	13.92	16.32
9	Central African Republic	836	1,639	1,185	1.74	3.22	2.12
10	Chad	4,057	5,735	4,291	3.07	3.68	2.42
11	Comoros	295	1,234	1,371	4.22	15.90	16.38
12	Congo	4,571	6,150	6,615	9.47	11.30	11.08
13	Cote d'Ivoire	12,631	20,623	26,650	5.62	8.09	9.46
14	DRC	61,368	107,427	118,355	8.35	12.34	11.95
15	Equatorial Guinea	-	406	1,401	-	2.70	8.37
16	Eritrea	4,262	5,016	5,074	12.93	14.56	13.77
17	Eswatini	3,735	4,720	5,256	33.40	40.67	43.74
18	Ethiopia	6,925	77,933	165,803	0.71	7.01	13.44
19	Gabon	437	6,818	5,672	2.30	31.10	23.74
20	Gambia	2,645	1,818	1,808	12.45	7.44	6.68
21	Ghana	48,691	135,649	148,086	17.69	43.94	44.24
22	Guinea	6,289	8,061	5,912	5.69	6.42	4.27
23	Guinea-Bissau	880	1,421	2,076	5.18	7.38	9.86
24	Kenya	40,292	59,901	109,796	9.00	11.99	20.32
25	Lesotho	-	9,645	4,014	-	43.88	17.41
26	Liberia	806	9,415	5,413	1.82	19.26	10.21
27	Madagascar	6,838	8,554	8,554	2.90	3.19	2.89
28	Malawi	3,593	7,957	10,208	2.24	4.33	5.00
29	Mali	6,218	8,460	8,247	3.66	4.24	3.65
30	Mauritania	4,214	4,921	7,843	11.26	11.52	16.56
31	Mauritius	3,963	5,553	5,166	30.70	42.87	39.75
32	Mozambique	10,697	14,174	30,552	4.24	4.82	9.27
33	Namibia	4,110	12,961	15,575	18.64	53.88	60.67
34	Niger	4,450	5,013	5,390	2.39	2.22	2.06
35	Nigeria	176,195	301,579	367,468	10.08	15.20	16.81
36	Rwanda	10,247	14,816	15,491	9.23	11.82	11.24
37	Sao Tome and Principe	473	456	465	24.41	21.58	20.46
38	Senegal	5,628	7,592	8,018	4.14	4.87	4.63
39	Seychelles	419	1,430	1,456	43.32	138.70	135.95
40	Sierra Leone	8,293	6,148	17,937	11.91	7.82	20.84
41	South Africa	58,722	287,458	397,261	10.90	50.13	66.33
42	South Sudan	-	5,438	7,550	-	5.23	6.92
43	Tanzania	20,800	31,940	31,940	4.22	5.50	4.88
44	Togo	2,136	3,251	4,494	3.01	4.04	5.08
45	Uganda	47,149	60,144	107,154	13.37	14.49	22.68
46	Zambia	2,773	24,178	59,177	1.82	13.56	29.56
47	Zimbabwe	18,722	28,728	56,138	13.81	19.09	34.40
48	**WHO AFRICAN REGION**	**652,548**	**1,468,265**	**2,033,359**	**9.12**	**17.67**	**19.37**

- Indicates NO DATA

Appendix Table 8: The trends of dentists and dental assistants & therapists' stock and density in WHO AFRICAN REGION between 2013 and 2022

		Dentists			Dental Assistants and Therapists			Dentists			Dental Assistants and Therapists		
**S No**	**Country**	**2013**	**2018**	**2022**	**2013**	**2018**	**2022**	**Density per 10,000 (2013)**	**Density per 10,000 (2018)**	**Density per 10,000 (2022)**	**Density per 10,000 (2013)**	**Density per 10,000 (2018)**	**Density per 10,000 (2022)**
1	Algeria	-	15,437	16,223	-	46	46	-	3.68	3.61	-	0.01	0.01
2	Angola	-	1,652	254	-	796	796	-	0.53	0.07	-	0.25	0.22
3	Benin	-	12	50	-	33	7	-	0.01	0.04	-	0.03	0.01
4	Botswana	55	96	99	-	27	28	0.25	0.39	0.38	-	0.11	0.10
5	Burkina Faso	38	91	40	-	185	173	0.02	0.04	0.02	-	0.09	0.08
6	Burundi	4	14	14	-	-	-	0.00	0.01	0.01	-	-	-
7	Cameroon	58	308	317	-	-	88	0.03	0.12	0.11	-	-	0.03
8	Cape-Verde	-	81	12	-	74	74	-	1.42	0.21	-	1.30	1.25
9	Central African Republic	-	10	3	-	7	7	-	0.02	0.00	-	0.01	0.01
10	Chad	-	3	21	-	11	11	-	0.00	0.01	-	0.01	0.01
11	Comoros	18	32	32	-	-	-	0.26	0.41	0.38	-	-	-
12	Congo	-	27	10	-	-	-	-	0.05	0.02	-	-	-
13	Cote d'Ivoire	490	506	536	120	-	28	0.22	0.20	0.19	0.05	-	0.01
14	DRC	-	404	451	-	-	-	-	0.05	0.05	-	-	-
15	Equatorial Guinea	-	-	-	-	-	-	-	-	-	-	-	-
16	Eritrea	-	186	67	-	164	180	-	0.54	0.18	-	0.48	0.49
17	Eswatini	-	74	50	-	46	40	-	0.64	0.42	-	0.40	0.33
18	Ethiopia	-	1,889	3,522	-	144	195	-	0.17	0.29	-	0.01	0.02
19	Gabon	-	40	44	-	3	67	-	0.18	0.18	-	0.01	0.28
20	Gambia	17	4	9	-	10	16	0.08	0.02	0.03	-	0.04	0.06
21	Ghana	-	713	244	-	227	488	-	0.23	0.07	-	0.07	0.15
22	Guinea	-	73	39	-	-	50	-	0.06	0.03	-	-	0.04
23	Guinea-Bissau	-	8	20	-	8	2	-	0.04	0.09	-	0.04	0.01
24	Kenya	1,045	1,300	1,344	-	464	987	0.23	0.26	0.25	-	0.09	0.18
25	Lesotho	-	159	84	-	120	63	-	0.72	0.37	-	0.55	0.27
26	Liberia	-	14	8	-	10	10	-	0.03	0.01	-	0.02	0.02
27	Madagascar	111	372	251	-	184	23	0.05	0.14	0.08	-	0.07	0.01
28	Malawi	-	112	101	-	76	173	-	0.06	0.05	-	0.04	0.08
29	Mali	-	30	30	-	53	53	-	0.02	0.01	-	0.03	0.02
30	Mauritania	-	151	149	-	-	-	-	0.35	0.31	-	-	-
31	Mauritius	351	466	414	-	65	78	2.72	3.60	3.19	-	0.50	0.60
32	Mozambique	136	255	348	238	340	341	0.05	0.09	0.11	0.09	0.12	0.10
33	Namibia	68	181	224	-	108	158	0.31	0.75	0.87	-	0.45	0.61
34	Niger	30	30	28	-	-	-	0.02	0.01	0.01	-	-	-
35	Nigeria	-	4,358	5,048	-	21,129	13,540	-	0.22	0.23	-	1.07	0.62
36	Rwanda	127	228	236	-	-	-	0.11	0.18	0.17	-	-	-
37	Sao Tome and Principe	-	6	6	-	-	-	-	0.28	0.26	-	-	-
38	Senegal	54	136	223	-	100	75	0.04	0.09	0.13	-	0.06	0.04
39	Seychelles	14	38	44	-	125	131	1.45	3.69	4.11	-	12.12	12.23
40	Sierra Leone	-	15	15	-	414	145	-	0.02	0.02	-	0.53	0.17
41	South Africa	5,770	6,433	1,976	4,625	661	1,550	1.07	1.12	0.33	0.86	0.12	0.26
42	South Sudan	-	32	32	-	22	128	-	0.03	0.03	-	0.02	0.12
43	Tanzania	99	682	755	366	768	582	0.02	0.12	0.12	0.07	0.13	0.09
44	Togo	18	21	22	-	25	1	0.03	0.03	0.02	-	0.03	0.00
45	Uganda	276	26	504	-	26	1,084	0.08	0.01	0.11	-	0.01	0.23
46	Zambia	-	160	306	282	412	743	-	0.09	0.15	0.19	0.23	0.37

		Dentists			Dental Assistants and Therapists			Dentists			Dental Assistants and Therapists		
**S No**	**Country**	**2013**	**2018**	**2022**	**2013**	**2018**	**2022**	**Density per 10,000 (2013)**	**Density per 10,000 (2018)**	**Density per 10,000 (2022)**	**Density per 10,000 (2013)**	**Density per 10,000 (2018)**	**Density per 10,000 (2022)**
47	Zimbabwe	236	142	201	171	508	206	0.17	0.09	0.12	0.13	0.34	0.13
48	**WHO AFRICAN REGION**	**9,015**	**37,007**	**34,405**	**5,802**	**27,391**	**22,367**	**0.15**	**0.44**	**0.37**	**0.03**	**0.41**	**0.41**

- Indicates NO DATA

Appendix Table 9: The trends of Pharmacists and Pharmaceutical Technicians and Assistants' stock and density in WHO AFRICAN REGION between 2013 and 2022

		Pharmacists			Pharmaceutical Technicians and Assistants			Pharmacists			Pharmaceutical Technicians and Assistants		
S No	Country	2013	2018	2022	2013	2018	2022	Density per 10,000 (2013)	Density per 10,000 (2018)	Density per 10,000 (2022)	Density per 10,000 (2013)	Density per 10,000 (2018)	Density per 10,000 (2022)
1	Algeria	-	18,807	13,642	-	1,536	1,536	-	4.49	3.04	-	0.37	0.34
2	Angola	-	2,302	1,434	-	1,605	943	-	0.74	0.40	-	0.51	0.27
3	Benin	1,330	306	382	770	45	56	1.29	0.26	0.29	0.75	0.04	0.04
4	Botswana	-	156	144	-	435	449	-	0.64	0.55	-	1.77	1.71
5	Burkina Faso	207	636	591	148	156	210	0.12	0.31	0.26	0.08	0.08	0.09
6	Burundi	4	119	124	-	-	6	0.00	0.10	0.10	-	-	0.00
7	Cameroon	162	350	602	1,178	-	369	0.07	0.14	0.22	0.54	-	0.13
8	Cape-Verde	-	172	29	-	92	7	-	3.01	0.48	-	1.61	0.13
9	Central African Republic	20	36	3	-	-	-	0.04	0.07	0.00	-	-	-
10	Chad	72	269	188	-	171	320	0.05	0.17	0.11	-	0.11	0.18
11	Comoros	16	36	40	-	19	71	0.23	0.46	0.48	-	0.24	0.85
12	Congo	-	157	32	-	84	92	-	0.29	0.05	-	0.15	0.15
13	Cote d'Ivoire	2,430	2,495	1,491	2,310	217	257	1.08	0.98	0.53	1.03	0.09	0.09
14	DRC	283	1,687	928	-	226	378	0.04	0.19	0.09	-	0.03	0.04
15	Equatorial Guinea	-	10	14	-	-	91	-	0.07	0.08	-	-	0.54
16	Eritrea	-	451	229	-	300	331	-	1.31	0.62	-	0.87	0.90
17	Eswatini	-	281	60	-	171	115	-	2.42	0.50	-	1.47	0.95
18	Ethiopia	-	10,752	22,344	-	6,845	13,620	-	0.97	1.81	-	0.62	1.10
19	Gabon	-	227	187	-	106	233	-	1.04	0.78	-	0.48	0.98
20	Gambia	5	78	4	99	76	94	0.02	0.32	0.02	0.47	0.31	0.35
21	Ghana	-	918	5,736	-	1,352	1,507	-	0.30	1.71	-	0.44	0.45
22	Guinea	142	255	255	-	-	-	0.13	0.20	0.18	-	-	-
23	Guinea-Bissau	-	13	2	-	69	80	-	0.07	0.01	-	0.36	0.38
24	Kenya	2,202	964	1,337	-	1,331	6,240	0.49	0.19	0.25	-	0.27	1.15
25	Lesotho	-	398	257	-	301	347	-	1.81	1.11	-	1.37	1.50
26	Liberia	-	109	170	-	962	575	-	0.22	0.32	-	1.97	1.08
27	Madagascar	17	319	30	-	10	10	0.01	0.12	0.01	-	0.00	0.00
28	Malawi	170	387	123	-	276	710	0.11	0.21	0.06	-	0.15	0.35
29	Mali	-	1,340	1,721	-	84	84	-	0.67	0.76	-	0.04	0.04
30	Mauritania	-	87	97	-	13	13	-	0.20	0.20	-	0.03	0.03
31	Mauritius	460	748	41	-	217	314	3.56	5.77	0.32	-	1.68	2.42
32	Mozambique	103	305	498	1,388	2,051	3,299	0.04	0.10	0.15	0.55	0.70	1.00
33	Namibia	-	598	655	-	309	402	-	2.49	2.55	-	1.28	1.57
34	Niger	51	60	49	-	-	4	0.03	0.03	0.02	-	-	0.00
35	Nigeria	20,035	24,668	21,006	-	5,793	2,673	1.15	1.24	0.96	-	0.29	0.12
36	Rwanda	114	886	886	-	201	201	0.10	0.71	0.64	-	0.16	0.15
37	Sao Tome and Principe	-	37	37	-	-	-	-	1.75	1.63	-	-	-
38	Senegal	143	162	352	371	53	57	0.11	0.10	0.20	0.27	0.03	0.03
39	Seychelles	4	124	70	-	63	63	0.41	12.03	6.54	-	6.11	5.88
40	Sierra Leone	-	156	193	-	21	857	-	0.20	0.22	-	0.03	1.00
41	South Africa	13,642	14,412	17,703	7,320	1,783	1,783	2.53	2.51	2.96	1.36	0.31	0.30
42	South Sudan	-	360	160	-	150	291	-	0.35	0.15	-	0.14	0.27
43	Tanzania	339	1,845	2,262	317	1,246	5,583	0.07	0.32	0.35	0.06	0.21	0.85
44	Togo	17	226	255	-	3	73	0.02	0.28	0.29	-	0.00	0.08
45	Uganda	550	324	1,712	650	234	1,820	0.16	0.08	0.36	0.18	0.06	0.39
46	Zambia	409	727	1,425	814	1,284	1,800	0.27	0.41	0.71	0.53	0.72	0.90

		Pharmacists			Pharmaceutical Technicians and Assistants			Pharmacists			Pharmaceutical Technicians and Assistants		
S No	Country	2013	2018	2022	2013	2018	2022	Density per 10,000 (2013)	Density per 10,000 (2018)	Density per 10,000 (2022)	Density per 10,000 (2013)	Density per 10,000 (2018)	Density per 10,000 (2022)
47	Zimbabwe	394	1,397	1,902	511	1,827	680	0.29	0.93	1.17	0.38	1.21	0.42
48	**WHO AFRICAN REGION**	**43,321**	**91,152**	**101,401**	**15,876**	**31,717**	**48,644**	**0.26**	**1.09**	**0.73**	**0.13**	**0.56**	**0.62**

- Indicates NO DATA

Appendix Table 10: The trends of community health workers' stock and density in WHO AFRICAN REGION between 2013 and 2022

SNo	Country	Community Health workers			Density per 10,000 (2013)	Density per 10,000 (2018)	Density per 10,000 (2022)
		2013	2018	2022			
1	Algeria	-	-	-	-	-	-
2	Angola	-	1,680	7	-	0.54	0.00
3	Benin	-	-	21	-	-	0.02
4	Botswana	166	-	-	0.75	-	-
5	Burkina Faso	2,099	2,749	3,145	1.19	1.35	1.39
6	Burundi	-	11,845	11,845	-	10.31	9.19
7	Cameroon	-	-	310	-	-	0.11
8	Cape-Verde	-	117	155	-	2.05	2.61
9	Central African Republic	-	115	1,595	-	0.23	2.86
10	Chad	-	7	8,077	-	0.00	4.56
11	Comoros	-	-	-	-	-	-
12	Congo	-	-	-	-	-	-
13	Cote d'Ivoire	-	14,556	14,556	-	5.71	5.17
14	DRC	-	-	-	-	-	-
15	Equatorial Guinea	-	-	146	-	-	0.87
16	Eritrea	-	243	2,626	-	0.71	7.13
17	Eswatini	-	6,324	4,586	-	54.50	38.17
18	Ethiopia	-	37,259	41,101	-	3.35	3.33
19	Gabon	-	117	109	-	0.53	0.46
20	Gambia	-	1,553	1,553	-	6.35	5.74
21	Ghana	5,293	15,820	15,820	1.92	5.12	4.73
22	Guinea	6,700	16,567	16,567	6.06	13.20	11.95
23	Guinea-Bissau	-	4,057	4,818	-	21.08	22.88
24	Kenya	-	58,079	86,423	-	11.63	16.00
25	Lesotho	-	14,508	14,508	-	66.00	62.92
26	Liberia	-	3,391	12,625	-	6.94	23.81
27	Madagascar	-	35,000	41,616	-	13.04	14.05
28	Malawi	-	10,016	10,747	-	5.45	5.27
29	Mali	-	1,152	3,303	-	0.58	1.46
30	Mauritania	-	500	500	-	1.17	1.06
31	Mauritius	-	180	916	-	1.39	7.05
32	Mozambique	1,204	4,787	6,992	0.48	1.63	2.12
33	Namibia	-	2,292	2,292	-	9.53	8.93
34	Niger	6,560	55	2,239	3.52	0.02	0.85
35	Nigeria	-	116,454	154,477	-	5.87	7.07
36	Rwanda	-	45,000	58,567	-	35.91	42.51
37	Sao Tome and Principe	-	-	-	-	-	-
38	Senegal	770	17,417	18,757	0.57	11.18	10.83
39	Seychelles	-	-	-	-	-	-
40	SierraLeone	-	505	13,966	-	0.64	16.23
41	South Africa	-	54,180	63,290	-	9.45	10.57
42	South Sudan	-	1,455	2,826	-	1.40	2.59
43	Tanzania	-	-	20,737	-	-	3.17
44	Togo	-	7,500	7,500	-	9.32	8.48
45	Uganda	179,175	179,000	179,000	50.80	43.12	37.88
46	Zambia	-	1,283	2,421	-	0.72	1.21
47	Zimbabwe	11,200	15,888	19,722	8.26	10.56	12.08
48	**WHO AFRICAN REGION**	**213,167**	**681,651**	**850,462**	**1.56**	**7.88**	**8.88**

- Indicates NO DATA

Appendix Table 11: The trends of dieticians and nutritionists' stock and density in WHO AFRICAN REGION between 2013 and 2022

SNo	Country	Dieticians and nutritionists			Density per 10,000 (2013)	Density per 10,000 (2018)	Density per 10,000 (2022)
		2013	2018	2022			
1	Algeria	1	381	8	0.00	0.09	0.00
2	Angola	85	4	197	0.03	0.00	0.06
3	Benin	-	8	21	-	0.01	0.02
4	Botswana	668	-	-	3.01	-	-
5	Burkina Faso	128	19	15	0.07	0.01	0.01
6	Burundi	44	10	10	0.04	0.01	0.01
7	Cameroon	-	38	38	-	0.02	0.01
8	Cape-Verde	3,781	18	7	70.03	0.32	0.13
9	Central African Republic	44	77	1,634	0.09	0.15	2.93
10	Chad	406	58	58	0.31	0.04	0.03
11	Comoros	-	-	-	-	-	-
12	Congo	819	-	582	1.70	-	0.97
13	Cote d'Ivoire	-	745	9	-	0.29	0.00
14	DRC	19	535	105	0.00	0.06	0.01
15	Equatorial Guinea	5	6	25	0.04	0.04	0.15
16	Eritrea	-	-	-	-	-	-
17	Eswatini	15	31	14	0.13	0.27	0.12
18	Ethiopia	50	1,385	1,385	0.01	0.12	0.11
19	Gabon	999	6	6	5.25	0.03	0.03
20	Gambia	-	-	59	-	-	0.22
21	Ghana	4	-	3,321	0.00	-	0.99
22	Guinea	-	-	-	-	-	-
23	Guinea-Bissau	496	-	-	2.92	-	-
24	Kenya	211	1,147	10,071	0.05	0.23	1.86
25	Lesotho	11	29	43	0.05	0.13	0.19
26	Liberia	-	-	-	-	-	-
27	Madagascar	-	414	414	-	0.15	0.14
28	Malawi	-	25	10	-	0.01	0.00
29	Mali	-	7	3,342	-	0.00	1.48
30	Mauritania	35	4,038	4,038	0.09	9.46	8.53
31	Mauritius	-	61	1	-	0.47	0.01
32	Mozambique	-	-	856	-	-	0.26
33	Namibia	-	33	33	-	0.14	0.13
34	Niger	-	42	42	-	0.02	0.02
35	Nigeria	-	7	77	-	0.00	0.00
36	Rwanda	-	-	207	-	-	0.15
37	Sao Tome and Principe	4	61	51	0.21	2.89	2.22
38	Senegal	-	4	4	-	0.00	0.00
39	Seychelles	-	5	5	-	0.48	0.47
40	SierraLeone	-	103	120	-	0.13	0.14
41	South Africa	-	-	-	-	-	-
42	South Sudan	-	22	22	-	0.02	0.02
43	Tanzania	-	855	84	-	0.15	0.01
44	Togo	-	12	12	-	0.01	0.01
45	Uganda	-	3	93	-	0.00	0.02
46	Zambia	-	404	774	-	0.23	0.39
47	Zimbabwe	845	-	280	0.62	-	0.17
48	**WHO AFRICAN REGION**	**8,670**	**10,593**	**28,074**	**1.80**	**0.34**	**0.47**

- Indicates NO DATA

Appendix Table 12: The trends of Environmental and occupational health & hygiene workers' stock and density in WHO AFRICAN REGION between 2013 and 2022

SNo	Country	Environmental and occupational health & hygiene workers			Density per 10,000 (2013)	Density per 10,000 (2018)	Density per 10,000 (2022)
		2013	2018	2022			
1	Algeria	199	1,249	23	0.05	0.30	0.01
2	Angola	517	657	15	0.20	0.21	0.00
3	Benin	3,830	619	1,570	3.72	0.52	1.18
4	Botswana	166	99	99	0.75	0.40	0.38
5	Burkina Faso	1,791	160	32	1.02	0.08	0.01
6	Burundi	-	372	186	-	0.32	0.14
7	Cameroon	-	238	7	-	0.09	0.00
8	Cape-Verde	3,881	3,207	439	71.88	56.14	7.40
9	Central African Republic	257	105	60	0.54	0.21	0.11
10	Chad	1,258	76	76	0.95	0.05	0.04
11	Comoros	755	389	389	10.80	5.01	4.65
12	Congo	4,104	374	374	8.50	0.69	0.63
13	Cote d'Ivoire	-	1,425	1,072	-	0.56	0.38
14	DRC	634	167	233	0.09	0.02	0.02
15	Equatorial Guinea	1	-	134	0.01	-	0.80
16	Eritrea	-	243	243	-	0.71	0.66
17	Eswatini	4,646	275	119	41.54	2.37	0.99
18	Ethiopia	461	2,662	5,410	0.05	0.24	0.44
19	Gabon	296	312	312	1.56	1.42	1.31
20	Gambia	-	316	2,305	-	1.29	8.52
21	Ghana	351	47	4,778	0.13	0.02	1.43
22	Guinea	-	363	363	-	0.29	0.26
23	Guinea-Bissau	-	3	3	-	0.02	0.01
24	Kenya	201	5,310	636	0.04	1.06	0.12
25	Lesotho	205	144	144	0.99	0.66	0.62
26	Liberia	2,036	285	285	4.60	0.58	0.54
27	Madagascar	174	15	254	0.07	0.01	0.09
28	Malawi	-	35	35	-	0.02	0.02
29	Mali	-	78	3,642	-	0.04	1.61
30	Mauritania	209	4,745	4,745	0.56	11.11	10.02
31	Mauritius	-	48	311	-	0.37	2.39
32	Mozambique	85	-	6	0.03	-	0.00
33	Namibia	-	218	261	-	0.91	1.01
34	Niger	-	101	101	-	0.04	0.04
35	Nigeria	-	14,645	14,645	-	0.74	0.67
36	Rwanda	-	-	226	-	-	0.16
37	Sao Tome and Principe	24	548	282	1.24	25.93	12.38
38	Senegal	3,518	1,925	2,494	2.59	1.24	1.44
39	Seychelles	-	50	50	-	4.85	4.67
40	SierraLeone	-	406	790	-	0.52	0.92
41	South Africa	-	3,585	3,585	-	0.63	0.60
42	South Sudan	-	250	250	-	0.24	0.23
43	Tanzania	3,255	1,905	1,204	0.66	0.33	0.18
44	Togo	-	340	340	-	0.42	0.38
45	Uganda	-	16	426	-	0.00	0.09
46	Zambia	-	2,016	4,392	-	1.13	2.19
47	Zimbabwe	1,885	1,644	2,684	1.39	1.09	1.64
48	**WHO AFRICAN REGION**	**34,739**	**51,667**	**60,028**	**3.28**	**2.61**	**1.52**

- Indicates NO DATA

Appendix Table 13: The trends of managerial staff stock and density in WHO AFRICAN REGION between 2013 and 2022

SNo	Country	Managerial staff			Density per 10,000 (2013)	Density per 10,000 (2018)	Density per 10,000 (2022)
		2013	2018	2022			
1	Algeria	-	10,838	10,838	-	2.58	2.41
2	Angola	-	328	2,072	-	0.10	0.58
3	Benin	-	-	-	-	-	-
4	Botswana	-	156	159	-	0.64	0.60
5	Burkina Faso	-	653	653	-	0.32	0.29
6	Burundi	-	1,383	1,383	-	1.20	1.07
7	Cameroon	-	250	1,195	-	0.10	0.43
8	Cape-Verde	-	61	61	-	1.07	1.03
9	Central African Republic	-	22	22	-	0.04	0.04
10	Chad	-	732	428	-	0.47	0.24
11	Comoros	-	-	-	-	-	-
12	Congo	-	790	790	-	1.45	1.32
13	Cote d'Ivoire	-	91	91	-	0.04	0.03
14	DRC	-	2,651	4,695	-	0.30	0.47
15	Equatorial Guinea	-	-	-	-	-	-
16	Eritrea	-	-	381	-	-	1.03
17	Eswatini	-	195	195	-	1.68	1.62
18	Ethiopia	-	4,365	4,365	-	0.39	0.35
19	Gabon	-	318	390	-	1.45	1.63
20	Gambia	-	246	522	-	1.01	1.93
21	Ghana	-	1,033	1,033	-	0.33	0.31
22	Guinea	-	189	189	-	0.15	0.14
23	Guinea-Bissau	-	130	130	-	0.68	0.62
24	Kenya	-	-	-	-	-	-
25	Lesotho	-	110	42	-	0.50	0.18
26	Liberia	-	1,404	1,404	-	2.87	2.65
27	Madagascar	-	411	411	-	0.15	0.14
28	Malawi	-	178	561	-	0.10	0.27
29	Mali	-	25	25	-	0.01	0.01
30	Mauritania	-	20	20	-	0.05	0.04
31	Mauritius	-	315	315	-	2.43	2.42
32	Mozambique	-	2,466	3,337	-	0.84	1.01
33	Namibia	-	313	77	-	1.30	0.30
34	Niger	-	226	226	-	0.10	0.09
35	Nigeria	-	-	-	-	-	-
36	Rwanda	-	-	-	-	-	-
37	Sao Tome and Principe	-	79	79	-	3.74	3.47
38	Senegal	-	135	188	-	0.09	0.11
39	Seychelles	-	-	-	-	-	-
40	SierraLeone	-	93	487	-	0.12	0.57
41	South Africa	-	-	26,692	-	-	4.46
42	South Sudan	-	20	20	-	0.02	0.02
43	Tanzania	-	633	633	-	0.11	0.10
44	Togo	-	63	1,377	-	0.08	1.56
45	Uganda	-	-	-	-	-	-
46	Zambia	-	-	-	-	-	-
47	Zimbabwe	229	414	414	0.17	0.28	0.25
48	**WHO AFRICAN REGION**	**229**	**31,336**	**65,901**	**0.00**	**0.57**	**0.72**

- Indicates NO DATA

Appendix Table 14: The trends of Medical and dental Prosthetic Technicians' stock and density in WHO AFRICAN REGION between 2013 and 2022

SNo	Country	Medical and dental Prosthetic Technicians			Density per 10,000 (2013)	Density per 10,000 (2018)	Density per 10,000 (2022)
		2013	2018	2022			
1	Algeria	-	836	836	-	0.20	0.19
2	Angola	-	75	75	-	0.02	0.02
3	Benin	-	15	15	-	0.01	0.01
4	Botswana	-	27	33	-	0.11	0.13
5	Burkina Faso	-	3	14	-	0.00	0.01
6	Burundi	-	-	-	-	-	-
7	Cameroon	-	-	19	-	-	0.01
8	Cape-Verde	-	20	20	-	0.35	0.34
9	Central African Republic	-	1	1	-	0.00	0.00
10	Chad	-	-	11	-	-	0.01
11	Comoros	-	-	-	-	-	-
12	Congo	-	-	-	-	-	-
13	Cote d'Ivoire	190	40	40	0.08	0.02	0.01
14	DRC	-	-	-	-	-	-
15	Equatorial Guinea	-	5	5	-	0.03	0.03
16	Eritrea	-	-	-	-	-	-
17	Eswatini	-	3	3	-	0.03	0.02
18	Ethiopia	-	52	130	-	0.00	0.01
19	Gabon	-	46	19	-	0.21	0.08
20	Gambia	3	7	6	0.01	0.03	0.02
21	Ghana	-	167	1,550	-	0.05	0.46
22	Guinea	-	-	-	-	-	-
23	Guinea-Bissau	-	5	2	-	0.03	0.01
24	Kenya	-	468	987	-	0.09	0.18
25	Lesotho	-	10	13	-	0.05	0.06
26	Liberia	-	-	-	-	-	-
27	Madagascar	-	52	13	-	0.02	0.00
28	Malawi	-	-	12	-	-	0.01
29	Mali	-	15	15	-	0.01	0.01
30	Mauritania	-	9	9	-	0.02	0.02
31	Mauritius	-	12	15	-	0.09	0.12
32	Mozambique	44	304	83	0.02	0.10	0.03
33	Namibia	-	470	15	-	1.95	0.06
34	Niger	-	11	7	-	0.00	0.00
35	Nigeria	-	6,165	322	-	0.31	0.01
36	Rwanda	23	-	-	0.02	-	-
37	Sao Tome and Principe	-	-	-	-	-	-
38	Senegal	-	100	18	-	0.06	0.01
39	Seychelles	-	2	11	-	0.19	1.03
40	SierraLeone	-	128	15	-	0.16	0.02
41	South Africa	-	3,330	482	-	0.58	0.08
42	South Sudan	-	21	21	-	0.02	0.02
43	Tanzania	60	89	16	0.01	0.02	0.00
44	Togo	-	55	23	-	0.07	0.03
45	Uganda	227	30	1,282	0.06	0.01	0.27
46	Zambia	53	-	28	0.03	-	0.01
47	Zimbabwe	26	40	318	0.02	0.03	0.19
48	**WHO AFRICAN REGION**	**626**	**12,613**	**6,484**	**0.01**	**0.10**	**0.07**

- Indicates NO DATA

Appendix Table 15: The trends of Medical and Pathology Laboratory scientists' stock and density in WHO AFRICAN REGION between 2013 and 2022

SNo	Country	Medical and Pathology Laboratory scientists			Density per 10,000 (2013)	Density per 10,000 (2018)	Density per 10,000 (2022)
		2013	2018	2022			
1	Algeria	-	136	136	-	0.03	0.03
2	Angola	-	468	13	-	0.15	0.00
3	Benin	-	668	668	-	0.56	0.50
4	Botswana	-	160	191	-	0.65	0.73
5	Burkina Faso	-	648	899	-	0.32	0.40
6	Burundi	-	1,071	1,071	-	0.93	0.83
7	Cameroon	-	241	293	-	0.10	0.10
8	Cape-Verde	-	65	65	-	1.14	1.10
9	Central African Republic	-	112	112	-	0.22	0.20
10	Chad	-	814	1,028	-	0.52	0.58
11	Comoros	-	161	161	-	2.07	1.92
12	Congo	-	320	320	-	0.59	0.54
13	Cote d'Ivoire	2,380	497	497	1.06	0.19	0.18
14	DRC	2,126	2,934	2,934	0.29	0.34	0.30
15	Equatorial Guinea	-	134	134	-	0.89	0.80
16	Eritrea	-	549	340	-	1.59	0.92
17	Eswatini	-	370	186	-	3.19	1.55
18	Ethiopia	-	10,450	23,757	-	0.94	1.93
19	Gabon	-	439	257	-	2.00	1.07
20	Gambia	11	138	18	0.05	0.56	0.07
21	Ghana	-	1,563	4,227	-	0.51	1.26
22	Guinea	-	181	181	-	0.14	0.13
23	Guinea-Bissau	-	53	1	-	0.28	0.01
24	Kenya	-	-	10,000	-	-	1.85
25	Lesotho	-	416	416	-	1.89	1.80
26	Liberia	-	115	115	-	0.24	0.22
27	Madagascar	-	306	306	-	0.11	0.10
28	Malawi	-	542	90	-	0.30	0.04
29	Mali	-	1,496	1,496	-	0.75	0.66
30	Mauritania	-	326	326	-	0.76	0.69
31	Mauritius	-	291	318	-	2.25	2.45
32	Mozambique	1,908	5,726	2,193	0.76	1.95	0.67
33	Namibia	-	1,739	97	-	7.23	0.38
34	Niger	-	432	432	-	0.19	0.16
35	Nigeria	-	26,677	36,833	-	1.34	1.69
36	Rwanda	-	1,990	1,990	-	1.59	1.44
37	Sao Tome and Principe	-	146	146	-	6.91	6.42
38	Senegal	-	1,045	508	-	0.67	0.29
39	Seychelles	-	71	71	-	6.89	6.63
40	SierraLeone	-	311	23	-	0.40	0.03
41	South Africa	144	8,415	19	0.03	1.47	0.00
42	South Sudan	-	272	635	-	0.26	0.58
43	Tanzania	89	4,361	1,552	0.02	0.75	0.24
44	Togo	407	857	539	0.57	1.07	0.61
45	Uganda	-	3,874	3,772	-	0.93	0.80
46	Zambia	184	18	641	0.12	0.01	0.32
47	Zimbabwe	343	644	757	0.25	0.43	0.46
48	**WHO AFRICAN REGION**	**7,592**	**82,242**	**100,765**	**0.07**	**1.20**	**0.93**

- Indicates NO DATA

Appendix Table 16: The trends of Medical and Pathology Laboratory Technicians' stock and density in WHO AFRICAN REGION between 2013 and 2022

SNo	Country	Medical and Pathology Laboratory Technicians			Density per 10,000 (2013)	Density per 10,000 (2018)	Density per 10,000 (2022)
		2013	2018	2022			
1	Algeria	-	15,238	15,238	-	3.63	3.39
2	Angola	-	98	18	-	0.03	0.01
3	Benin	830	241	1,602	0.81	0.20	1.20
4	Botswana	-	460	365	-	1.88	1.39
5	Burkina Faso	458	699	699	0.26	0.34	0.31
6	Burundi	-	1,071	911	-	0.93	0.71
7	Cameroon	-	5,235	239	-	2.09	0.09
8	Cape-Verde	-	65	65	-	1.14	1.10
9	Central African Republic	-	112	60	-	0.22	0.11
10	Chad	-	749	1,028	-	0.48	0.58
11	Comoros	86	8	16	1.23	0.10	0.19
12	Congo	-	320	777	-	0.59	1.30
13	Cote d'Ivoire	1,600	-	-	0.71	-	-
14	DRC	-	2,934	2,934	-	0.34	0.30
15	Equatorial Guinea	-	2	228	-	0.01	1.36
16	Eritrea	-	549	307	-	1.59	0.83
17	Eswatini	-	370	357	-	3.19	2.97
18	Ethiopia	-	10,450	13,915	-	0.94	1.13
19	Gabon	-	439	253	-	2.00	1.06
20	Gambia	141	138	138	0.66	0.56	0.51
21	Ghana	3,987	1,563	3,748	1.45	0.51	1.12
22	Guinea	-	181	181	-	0.14	0.13
23	Guinea-Bissau	-	172	197	-	0.89	0.94
24	Kenya	-	-	1,200	-	-	0.22
25	Lesotho	-	205	273	-	0.93	1.18
26	Liberia	-	-	-	-	-	-
27	Madagascar	-	306	306	-	0.11	0.10
28	Malawi	-	542	209	-	0.30	0.10
29	Mali	-	-	-	-	-	-
30	Mauritania	-	122	122	-	0.29	0.26
31	Mauritius	-	291	264	-	2.25	2.03
32	Mozambique	1,465	1,951	2,040	0.58	0.66	0.62
33	Namibia	-	510	1,065	-	2.12	4.15
34	Niger	-	432	480	-	0.19	0.18
35	Nigeria	-	311,269	44,492	-	15.69	2.04
36	Rwanda	1,545	1,990	1,990	1.39	1.59	1.44
37	Sao Tome and Principe	-	-	-	-	-	-
38	Senegal	546	328	295	0.40	0.21	0.17
39	Seychelles	-	71	71	-	6.89	6.63
40	SierraLeone	-	311	342	-	0.40	0.40
41	South Africa	273	5,731	6,353	0.05	1.00	1.06
42	South Sudan	-	272	272	-	0.26	0.25
43	Tanzania	1,997	4,361	4,743	0.41	0.75	0.72
44	Togo	-	615	887	-	0.76	1.00
45	Uganda	2,447	3,874	13,380	0.69	0.93	2.83
46	Zambia	1,126	1,712	2,235	0.74	0.96	1.12
47	Zimbabwe	519	1,126	919	0.38	0.75	0.56
48	**WHO AFRICAN REGION**	**17,020**	**377,113**	**125,216**	**0.21**	**1.25**	**1.02**

- Indicates NO DATA

Appendix Table 17: The trends of Medical Imaging and Therapeutic Equipment Technicians' stock and density in WHO AFRICAN REGION between 2013 and 2022

SNo	Country	Medical Imaging and Therapeutic Equipment Technicians			Density per 10,000 (2013)	Density per 10,000 (2018)	Density per 10,000 (2022)
		2013	2018	2022			
1	Algeria	-	6,245	6,245	-	1.49	1.39
2	Angola	-	789	159	-	0.25	0.04
3	Benin	206	137	352	0.20	0.11	0.26
4	Botswana	-	134	94	-	0.55	0.36
5	Burkina Faso	109	128	209	0.06	0.06	0.09
6	Burundi	-	86	101	-	0.07	0.08
7	Cameroon	-	124	4,864	-	0.05	1.74
8	Cape-Verde	-	27	27	-	0.47	0.46
9	Central African Republic	-	3	3	-	0.01	0.01
10	Chad	-	25	48	-	0.02	0.03
11	Comoros	-	498	29	-	6.41	0.34
12	Congo	-	97	97	-	0.18	0.16
13	Cote d'Ivoire	820	3,103	3,103	0.36	1.22	1.10
14	DRC	287	338	528	0.04	0.04	0.05
15	Equatorial Guinea	-	-	-	-	-	-
16	Eritrea	-	113	141	-	0.33	0.38
17	Eswatini	-	47	64	-	0.41	0.53
18	Ethiopia	-	1,041	2,513	-	0.09	0.20
19	Gabon	-	93	478	-	0.42	2.00
20	Gambia	32	149	172	0.15	0.61	0.64
21	Ghana	-	1,476	2,703	-	0.48	0.81
22	Guinea	-	-	-	-	-	-
23	Guinea-Bissau	-	17	51	-	0.09	0.24
24	Kenya	-	430	717	-	0.09	0.13
25	Lesotho	-	41	76	-	0.19	0.33
26	Liberia	-	9	9	-	0.02	0.02
27	Madagascar	-	214	214	-	0.08	0.07
28	Malawi	-	44	610	-	0.02	0.30
29	Mali	-	-	-	-	-	-
30	Mauritania	-	-	-	-	-	-
31	Mauritius	-	213	213	-	1.64	1.64
32	Mozambique	238	206	276	0.09	0.07	0.08
33	Namibia	-	303	438	-	1.26	1.71
34	Niger	-	82	82	-	0.04	0.03
35	Nigeria	-	2,648	4,262	-	0.13	0.20
36	Rwanda	116	152	152	0.10	0.12	0.11
37	Sao Tome and Principe	-	-	-	-	-	-
38	Senegal	22	542	277	0.02	0.35	0.16
39	Seychelles	-	7	7	-	0.68	0.65
40	SierraLeone	-	22	50	-	0.03	0.06
41	South Africa	8,476	8,878	9,281	1.57	1.55	1.55
42	South Sudan	-	6	16	-	0.01	0.01
43	Tanzania	223	681	703	0.05	0.12	0.11
44	Togo	-	148	115	-	0.18	0.13
45	Uganda	121	42	1,249	0.03	0.01	0.26
46	Zambia	500	761	2,633	0.33	0.43	1.32
47	Zimbabwe	239	480	435	0.18	0.32	0.27
48	**WHO AFRICAN REGION**	**11,389**	**30,579**	**43,796**	**0.07**	**0.44**	**0.43**

- Indicates NO DATA

Appendix Table 18: The trends of Medical Records and Health Information Technicians' stock and density in WHO AFRICAN REGION between 2013 and 2022

SNo	Country	Medical Records and Health Information Technicians 2013	2018	2022	Density per 10,000 (2013)	Density per 10,000 (2018)	Density per 10,000 (2022)
1	Algeria	-	610	610	-	0.15	0.14
2	Angola	-	190	174	-	0.06	0.05
3	Benin	-	-	-	-	-	-
4	Botswana	-	73	74	-	0.30	0.28
5	Burkina Faso	-	4	44	-	0.00	0.02
6	Burundi	-	108	108	-	0.09	0.08
7	Cameroon	-	-	85	-	-	0.03
8	Cape-Verde	-	14	14	-	0.25	0.24
9	Central African Republic	-	20	20	-	0.04	0.04
10	Chad	-	6	6	-	0.00	0.00
11	Comoros	-	-	-	-	-	-
12	Congo	-	-	-	-	-	-
13	Cote d'Ivoire	-	123	123	-	0.05	0.04
14	DRC	-	-	-	-	-	-
15	Equatorial Guinea	-	-	-	-	-	-
16	Eritrea	-	-	-	-	-	-
17	Eswatini	-	2,084	2,084	-	17.96	17.34
18	Ethiopia	-	2,953	2,953	-	0.27	0.24
19	Gabon	-	127	1	-	0.58	0.00
20	Gambia	-	-	-	-	-	-
21	Ghana	-	3,883	4,289	-	1.26	1.28
22	Guinea	-	66	66	-	0.05	0.05
23	Guinea-Bissau	-	24	24	-	0.12	0.11
24	Kenya	-	2,419	2,419	-	0.48	0.45
25	Lesotho	-	349	349	-	1.59	1.51
26	Liberia	-	65	65	-	0.13	0.12
27	Madagascar	-	11	11	-	0.00	0.00
28	Malawi	-	306	306	-	0.17	0.15
29	Mali	-	-	-	-	-	-
30	Mauritania	-	15	15	-	0.04	0.03
31	Mauritius	-	416	446	-	3.21	3.43
32	Mozambique	-	59	172	-	0.02	0.05
33	Namibia	-	37	37	-	0.15	0.14
34	Niger	-	22	22	-	0.01	0.01
35	Nigeria	-	34,393	31,315	-	1.73	1.43
36	Rwanda	-	-	-	-	-	-
37	Sao Tome and Principe	-	-	-	-	-	-
38	Senegal	-	17	2	-	0.01	0.00
39	Seychelles	-	-	-	-	-	-
40	SierraLeone	-	64	195	-	0.08	0.23
41	South Africa	-	-	-	-	-	-
42	South Sudan	-	57	1,006	-	0.05	0.92
43	Tanzania	470	724	724	0.10	0.12	0.11
44	Togo	-	-	-	-	-	-
45	Uganda	-	-	-	-	-	-
46	Zambia	-	1,232	1,232	-	0.69	0.62
47	Zimbabwe	-	718	718	-	0.48	0.44
48	**WHO AFRICAN REGION**	**470**	**51,189**	**49,709**	**0.00**	**0.64**	**0.63**

- Indicates NO DATA

Appendix Table 19: The trends of Optometrists and Ophthalmic Opticians' stock and density in WHO AFRICAN REGION between 2013 and 2022

SNo	Country	Optometrists and Ophthalmic Opticians			Density per 10,000 (2013)	Density per 10,000 (2018)	Density per 10,000 (2022)
		2013	2018	2022			
1	Algeria	-	1,533	1,533	-	0.37	0.34
2	Angola	-	56	9	-	0.02	0.00
3	Benin	-	-	-	-	-	-
4	Botswana	-	5	5	-	0.02	0.02
5	Burkina Faso	-	3	2	-	0.00	0.00
6	Burundi	-	-	-	-	-	-
7	Cameroon	-	-	10	-	-	0.00
8	Cape-Verde	-	40	5	-	0.70	0.08
9	Central African Republic	-	-	-	-	-	-
10	Chad	-	6	1	-	0.00	0.00
11	Comoros	-	-	-	-	-	-
12	Congo	-	-	-	-	-	-
13	Cote d'Ivoire	70	700	700	0.03	0.27	0.25
14	DRC	-	-	-	-	-	-
15	Equatorial Guinea	-	-	-	-	-	-
16	Eritrea	-	86	96	-	0.25	0.26
17	Eswatini	-	12	9	-	0.10	0.07
18	Ethiopia	-	330	674	-	0.03	0.05
19	Gabon	-	5	9	-	0.02	0.04
20	Gambia	17	16	18	0.08	0.07	0.07
21	Ghana	-	143	309	-	0.05	0.09
22	Guinea	-	-	-	-	-	-
23	Guinea-Bissau	-	16	12	-	0.08	0.06
24	Kenya	-	-	-	-	-	-
25	Lesotho	-	8	13	-	0.04	0.06
26	Liberia	-	5	5	-	0.01	0.01
27	Madagascar	-	56	56	-	0.02	0.02
28	Malawi	-	13	73	-	0.01	0.04
29	Mali	-	-	-	-	-	-
30	Mauritania	-	-	-	-	-	-
31	Mauritius	-	-	6	-	-	0.05
32	Mozambique	115	150	154	0.05	0.05	0.05
33	Namibia	-	147	162	-	0.61	0.63
34	Niger	-	-	-	-	-	-
35	Nigeria	-	5,371	5,632	-	0.27	0.26
36	Rwanda	-	13	13	-	0.01	0.01
37	Sao Tome and Principe	-	-	-	-	-	-
38	Senegal	-	108	125	-	0.07	0.07
39	Seychelles	-	28	25	-	2.72	2.36
40	SierraLeone	-	13	2	-	0.02	0.00
41	South Africa	3,588	3,848	374	0.67	0.67	0.06
42	South Sudan	-	7	24	-	0.01	0.02
43	Tanzania	82	213	260	0.02	0.04	0.04
44	Togo	-	5	2	-	0.01	0.00
45	Uganda	-	9	283	-	0.00	0.06
46	Zambia	18	61	244	0.01	0.03	0.12
47	Zimbabwe	-	20	20	-	0.01	0.01
48	**WHO AFRICAN REGION**	**3,890**	**13,026**	**10,866**	**0.02**	**0.14**	**0.11**

- Indicates NO DATA

Appendix Table 20: The trends of Other non-medical professional staff stock and density in WHO AFRICAN REGION between 2013 and 2022

SNo	Country	Other non-medical professional staff			Density per 10,000 (2013)	Density per 10,000 (2018)	Density per 10,000 (2022)
		2013	2018	2022			
1	Algeria	-	76,532	76,532	-	18.25	17.04
2	Angola	-	28,324	28,324	-	9.06	7.96
3	Benin	-	156	156	-	0.13	0.12
4	Botswana	-	1,248	1,579	-	5.09	6.00
5	Burkina Faso	-	871	23	-	0.43	0.01
6	Burundi	-	2,906	7,805	-	2.53	6.06
7	Cameroon	-	-	5	-	-	0.00
8	Cape-Verde	-	1,357	1,357	-	23.76	22.88
9	Central African Republic	-	65	65	-	0.13	0.12
10	Chad	-	436	7	-	0.28	0.00
11	Comoros	-	44	44	-	0.57	0.53
12	Congo	-	111	111	-	0.20	0.19
13	Cote d'Ivoire	-	139	139	-	0.05	0.05
14	DRC	-	74,310	2,076	-	8.53	0.21
15	Equatorial Guinea	-	-	-	-	-	-
16	Eritrea	-	3,563	3,563	-	10.34	9.67
17	Eswatini	-	1,215	75	-	10.47	0.62
18	Ethiopia	-	73,174	73,174	-	6.58	5.93
19	Gabon	-	1,939	1,052	-	8.85	4.40
20	Gambia	-	-	-	-	-	-
21	Ghana	-	23,627	23,627	-	7.65	7.06
22	Guinea	-	481	481	-	0.38	0.35
23	Guinea-Bissau	-	481	12	-	2.50	0.06
24	Kenya	-	2,759	3,295	-	0.55	0.61
25	Lesotho	-	2,130	792	-	9.69	3.44
26	Liberia	-	4,311	4,311	-	8.82	8.13
27	Madagascar	-	6,355	6,355	-	2.37	2.15
28	Malawi	-	-	-	-	-	-
29	Mali	-	8,306	8,306	-	4.17	3.68
30	Mauritania	-	1,532	1,532	-	3.59	3.23
31	Mauritius	-	3,295	3,295	-	25.44	25.36
32	Mozambique	-	26,735	800	-	9.09	0.24
33	Namibia	-	2,157	2,157	-	8.97	8.40
34	Niger	-	542	542	-	0.24	0.21
35	Nigeria	-	-	-	-	-	-
36	Rwanda	-	-	-	-	-	-
37	Sao Tome and Principe	-	-	-	-	-	-
38	Senegal	-	5,868	19	-	3.77	0.01
39	Seychelles	-	-	-	-	-	-
40	SierraLeone	-	-	-	-	-	-
41	South Africa	-	-	15,459	-	-	2.58
42	South Sudan	-	-	-	-	-	-
43	Tanzania	-	-	-	-	-	-
44	Togo	-	4,010	2,586	-	4.98	2.92
45	Uganda	-	-	-	-	-	-
46	Zambia	-	17,801	17,801	-	9.98	8.89
47	Zimbabwe	-	9,502	9,502	-	6.31	5.82
48	**WHO AFRICAN REGION**	**-**	**386,282**	**296,957**	**-**	**4.55**	**3.51**

- Indicates NO DATA

Appendix Table 21: The trends of Other non-medical support staff stock and density in WHO AFRICAN REGION between 2013 and 2022

SNo	Country	Other non-medical support staff			Density per 10,000 (2013)	Density per 10,000 (2018)	Density per 10,000 (2022)
		2013	2018	2022			
1	Algeria	-	75,033	75,033	-	17.90	16.71
2	Angola	-	-	8,431	-	-	2.37
3	Benin	-	1,994	1,994	-	1.67	1.49
4	Botswana	-	3,342	3,682	-	13.63	14.00
5	Burkina Faso	-	44	2,079	-	0.02	0.92
6	Burundi	-	9,003	9,003	-	7.83	6.98
7	Cameroon	-	-	74	-	-	0.03
8	Cape-Verde	-	95	95	-	1.66	1.60
9	Central African Republic	-	14	14	-	0.03	0.03
10	Chad	-	90	791	-	0.06	0.45
11	Comoros	-	115	115	-	1.48	1.37
12	Congo	-	445	445	-	0.82	0.75
13	Cote d'Ivoire	-	14	3,470	-	0.01	1.23
14	DRC	-	1,608	1,608	-	0.18	0.16
15	Equatorial Guinea	-	-	-	-	-	-
16	Eritrea	-	-	-	-	-	-
17	Eswatini	-	85	1,861	-	0.73	15.49
18	Ethiopia	-	2,872	2,872	-	0.26	0.23
19	Gabon	-	62	2,714	-	0.28	11.36
20	Gambia	-	-	2,193	-	-	8.10
21	Ghana	-	1,877	1,877	-	0.61	0.56
22	Guinea	-	-	-	-	-	-
23	Guinea-Bissau	-	69	78	-	0.36	0.37
24	Kenya	-	-	11,129	-	-	2.06
25	Lesotho	-	58	58	-	0.26	0.25
26	Liberia	-	3,601	3,601	-	7.36	6.79
27	Madagascar	-	-	-	-	-	-
28	Malawi	-	-	11,178	-	-	5.48
29	Mali	-	-	-	-	-	-
30	Mauritania	-	4	4	-	0.01	0.01
31	Mauritius	-	-	-	-	-	-
32	Mozambique	-	26,735	14,092	-	9.09	4.27
33	Namibia	-	-	-	-	-	-
34	Niger	-	462	462	-	0.20	0.18
35	Nigeria	-	3,919	3,919	-	0.20	0.18
36	Rwanda	-	-	-	-	-	-
37	Sao Tome and Principe	-	271	271	-	12.82	11.92
38	Senegal	-	1,722	403	-	1.11	0.23
39	Seychelles	-	-	-	-	-	-
40	SierraLeone	-	-	-	-	-	-
41	South Africa	-	15,266	15,266	-	2.66	2.55
42	South Sudan	-	-	-	-	-	-
43	Tanzania	-	3,630	3	-	0.62	0.00
44	Togo	-	1,554	1,657	-	1.93	1.87
45	Uganda	-	-	-	-	-	-
46	Zambia	-	-	-	-	-	-
47	Zimbabwe	-	1,122	1,122	-	0.75	0.69
48	**WHO AFRICAN REGION**	**-**	**155,106**	**181,593**	**-**	**1.80**	**2.57**

\- Indicates NO DATA

Appendix Table 22: The trends of Paramedical Practitioners' stock and density in WHO AFRICAN REGION between 2013 and 2022

SNo	Country	Paramedical Practitioners			Density per 10,000 (2013)	Density per 10,000 (2018)	Density per 10,000 (2022)
		2013	2018	2022			
1	Algeria	-	365	128,823	-	0.09	28.69
2	Angola	-	56	56	-	0.02	0.02
3	Benin	-	5,086	476	-	4.26	0.36
4	Botswana	-	58	7	-	0.24	0.03
5	Burkina Faso	-	-	1,698	-	-	0.75
6	Burundi	-	-	-	-	-	-
7	Cameroon	-	-	34	-	-	0.01
8	Cape-Verde	-	-	-	-	-	-
9	Central African Republic	-	200	200	-	0.39	0.36
10	Chad	-	-	-	-	-	-
11	Comoros	-	-	-	-	-	-
12	Congo	-	-	-	-	-	-
13	Cote d'Ivoire	-	21,440	5,000	-	8.41	1.78
14	DRC	-	-	-	-	-	-
15	Equatorial Guinea	-	-	101	-	-	0.60
16	Eritrea	-	-	-	-	-	-
17	Eswatini	-	29	29	-	0.25	0.24
18	Ethiopia	-	1,285	1,230	-	0.12	0.10
19	Gabon	-	58	84	-	0.26	0.35
20	Gambia	-	39	39	-	0.16	0.14
21	Ghana	-	2,116	2,656	-	0.69	0.79
22	Guinea	-	62	62	-	0.05	0.04
23	Guinea-Bissau	-	8	2	-	0.04	0.01
24	Kenya	13,216	21,942	25,400	2.95	4.39	4.70
25	Lesotho	-	4	3	-	0.02	0.01
26	Liberia	-	-	-	-	-	-
27	Madagascar	-	-	-	-	-	-
28	Malawi	-	98	41	-	0.05	0.02
29	Mali	-	-	-	-	-	-
30	Mauritania	-	65	65	-	0.15	0.14
31	Mauritius	-	24	24	-	0.19	0.18
32	Mozambique	-	6,281	6,873	-	2.13	2.08
33	Namibia	-	1,053	124	-	4.38	0.48
34	Niger	-	598	1,592	-	0.26	0.61
35	Nigeria	-	30	30	-	0.00	0.00
36	Rwanda	-	-	-	-	-	-
37	Sao Tome and Principe	-	-	-	-	-	-
38	Senegal	11	505	1,083	0.01	0.32	0.63
39	Seychelles	-	-	-	-	-	-
40	SierraLeone	-	319	650	-	0.41	0.76
41	South Africa	217	8,919	20,327	0.04	1.56	3.39
42	South Sudan	-	580	623	-	0.56	0.57
43	Tanzania	8,787	1,963	71,119	1.78	0.34	10.86
44	Togo	-	978	471	-	1.22	0.53
45	Uganda	-	-	-	-	-	-
46	Zambia	3,141	3,616	1,115	2.06	2.03	0.56
47	Zimbabwe	145	1,094	1,094	0.11	0.73	0.67
48	**WHO AFRICAN REGION**	**25,517**	**78,871**	**271,132**	**0.15**	**0.72**	**1.29**

- Indicates NO DATA

Appendix Table 23: The trends of Physiotherapists and physiotherapy assistants' stock and density in WHO AFRICAN REGION between 2013 and 2022

SNo	Country	Physiotherapists and physiotherapy assistants			Density per 10,000 (2013)	Density per 10,000 (2018)	Density per 10,000 (2022)
		2013	2018	2022			
1	Algeria	-	1,971	1,971	-	0.47	0.44
2	Angola	-	1,156	1,195	-	0.37	0.34
3	Benin	-	58	187	-	0.05	0.14
4	Botswana	-	42	34	-	0.17	0.13
5	Burkina Faso	-	18	47	-	0.01	0.02
6	Burundi	-	9	9	-	0.01	0.01
7	Cameroon	-	-	191	-	-	0.07
8	Cape-Verde	-	58	12	-	1.02	0.21
9	Central African Republic	-	-	155	-	-	0.28
10	Chad	-	2	18	-	0.00	0.01
11	Comoros	-	-	-	-	-	-
12	Congo	-	-	-	-	-	-
13	Cote d'Ivoire	60	151	122	0.03	0.06	0.04
14	DRC	-	395	793	-	0.05	0.08
15	Equatorial Guinea	-	-	-	-	-	-
16	Eritrea	-	137	149	-	0.40	0.41
17	Eswatini	-	29	32	-	0.25	0.27
18	Ethiopia	-	258	3,275	-	0.02	0.27
19	Gabon	-	43	2	-	0.20	0.01
20	Gambia	20	23	24	0.09	0.09	0.09
21	Ghana	-	304	381	-	0.10	0.11
22	Guinea	-	-	-	-	-	-
23	Guinea-Bissau	-	15	6	-	0.08	0.03
24	Kenya	-	-	631	-	-	0.12
25	Lesotho	-	22	9	-	0.10	0.04
26	Liberia	-	3	3	-	0.01	0.01
27	Madagascar	-	99	99	-	0.04	0.03
28	Malawi	-	52	248	-	0.03	0.12
29	Mali	-	-	-	-	-	-
30	Mauritania	-	7	7	-	0.02	0.01
31	Mauritius	-	64	64	-	0.49	0.49
32	Mozambique	287	377	553	0.11	0.13	0.17
33	Namibia	-	233	235	-	0.97	0.91
34	Niger	-	-	-	-	-	-
35	Nigeria	-	15,823	7,446	-	0.80	0.34
36	Rwanda	125	225	225	0.11	0.18	0.16
37	Sao Tome and Principe	-	-	-	-	-	-
38	Senegal	11	-	94	0.01	-	0.05
39	Seychelles	-	33	60	-	3.20	5.60
40	SierraLeone	-	13	7	-	0.02	0.01
41	South Africa	8,185	11,975	2,279	1.52	2.09	0.38
42	South Sudan	-	9	17	-	0.01	0.02
43	Tanzania	130	103	1,358	0.03	0.02	0.21
44	Togo	231	252	221	0.33	0.31	0.25
45	Uganda	114	18	572	0.03	0.00	0.12
46	Zambia	485	739	1,239	0.32	0.41	0.62
47	Zimbabwe	419	688	957	0.31	0.46	0.59
48	**WHO AFRICAN REGION**	**10,067**	**35,404**	**24,929**	**0.06**	**0.27**	**0.28**

\- Indicates NO DATA

Appendix Table 24: The trends of SDG 3c occupations versus other workers stock in WHO AFRICAN REGION between 2013 and 2022

		SDG 3c occupations workforce*			Other workers			Total		
S No	Country	2013	2018	2022	2013	2018	2022	2013	2018	2022
1	Algeria	11,139	175,647	220,805	200	240,105	366,964	11,339	415,752	587,769
2	Angola	30	60,468	78,870	602	50,395	58,067	632	110,863	136,937
3	Benin	7,923	5,769	11,051	15,066	11,707	13,973	22,989	17,476	25,024
4	Botswana	8,191	9,284	9,739	1,000	7,514	8,210	9,191	16,798	17,949
5	Burkina Faso	8,983	19,646	27,591	4,585	7,601	10,576	13,568	27,247	38,168
6	Burundi	7,170	10,533	10,709	44	37,621	42,120	7,214	48,154	52,830
7	Cameroon	22,321	14,532	23,282	-	16,843	8,354	22,321	31,375	31,636
8	Cape-Verde	711	1,666	3,734	7,662	5,297	2,476	8,373	6,963	6,210
9	Central African Republic	1,180	2,027	1,344	384	1,029	4,354	1,564	3,056	5,698
10	Chad	4,702	6,992	6,339	1,664	3,800	33,924	6,366	10,792	40,262
11	Comoros	452	1,541	1,868	841	1,215	754	1,293	2,756	2,622
12	Congo	4,571	6,962	7,792	4,923	3,307	4,346	9,494	10,269	12,138
13	Cote d'Ivoire	23,221	29,443	35,290	5,120	51,343	37,741	28,341	80,786	73,031
14	DRC	68,069	141,290	141,402	3,066	85,872	15,905	71,135	227,162	157,308
15	Equatorial Guinea	-	662	1,781	6	1,994	2,902	6	2,656	4,683
16	Eritrea	4,434	6,397	6,180	-	5,483	7,859	4,434	11,880	14,039
17	Eswatini	3,735	5,570	6,208	4,661	12,031	10,642	8,396	17,601	16,850
18	Ethiopia	6,925	108,826	229,225	511	160,072	188,294	7,436	268,898	417,519
19	Gabon	437	8,562	7,468	1,295	4,369	6,195	1,732	12,931	13,663
20	Gambia	2,979	2,284	2,205	1,007	2,625	7,047	3,986	4,909	9,253
21	Ghana	51,421	143,265	160,787	24,391	62,817	77,240	75,812	206,082	238,027
22	Guinea	8,275	11,176	9,201	6,700	18,091	18,091	14,975	29,267	27,292
23	Guinea-Bissau	1,234	2,097	2,712	496	6,168	6,479	1,730	8,265	9,191
24	Kenya	52,221	72,002	132,496	13,628	92,554	153,550	65,849	164,556	286,046
25	Lesotho	-	11,621	5,302	216	25,051	23,691	216	36,672	28,993
26	Liberia	974	11,203	7,129	2,036	13,864	23,098	3,010	25,067	30,227
27	Madagascar	10,489	14,669	14,098	174	44,152	50,968	10,663	58,821	65,067
28	Malawi	3,963	11,568	12,418	-	14,833	26,224	3,963	26,401	38,642
29	Mali	8,189	12,833	14,537	-	14,277	23,767	8,189	27,110	38,304
30	Mauritania	4,836	6,798	9,375	244	11,463	11,463	5,080	18,261	20,838
31	Mauritius	6,820	9,444	7,847	-	6,377	7,398	6,820	15,821	15,245
32	Mozambique	14,014	19,598	40,757	5,346	76,726	139,367	19,360	96,324	180,124
33	Namibia	4,178	15,602	18,611	-	10,043	8,448	4,178	25,645	27,059
34	Niger	5,477	6,168	6,177	6,560	3,596	6,818	12,037	9,764	12,995
35	Nigeria	261,989	441,434	495,916	331,077	537,756	303,917	593,066	979,190	799,833
36	Rwanda	11,592	17,779	18,462	2,695	49,680	63,680	14,287	67,459	82,142
37	Sao Tome and Principe	536	559	613	28	1,105	828	564	1,664	1,441
38	Senegal	6,196	10,926	11,285	4,932	34,349	29,940	11,128	45,275	41,225
39	Seychelles	530	2,020	2,592	-	525	594	530	2,545	3,187
40	Sierra Leone	8,293	7,320	20,253	4,557	2,288	18,899	12,850	9,608	39,151
41	South Africa	90,079	354,250	468,294	23,469	214,207	191,863	113,548	568,457	660,157
42	South Sudan	-	6,413	8,617	-	10,147	12,908	-	16,560	21,525
43	Tanzania	23,402	39,366	49,815	15,093	69,773	138,070	38,495	109,139	187,885
44	Togo	2,532	4,137	5,575	638	22,503	21,202	3,170	26,640	26,777
45	Uganda	51,995	77,940	121,326	207,921	190,898	215,144	259,916	268,838	336,470
46	Zambia	4,278	30,782	69,982	5,507	32,379	42,024	9,785	63,161	112,006
47	Zimbabwe	21,148	35,628	62,258	15,850	36,886	42,581	36,998	72,514	104,840

		SDG 3c occupations workforce*			Other workers			Total		
S No	Country	2013	2018	2022	2013	2018	2022	2013	2018	2022
48	WHO AFRICAN REGION	841,834	1,994,699	2,609,321	724,195	2,312,731	2,488,958	1,566,029	4,307,430	5,098,278

SDG 3c occupations* - Doctors, Nurses, midwives, pharmacists and Dentists

- Indicates NO DATA

Appendix Table 25: The trends of SDG 3c occupations versus other workers' density in WHO AFRICAN REGION between 2013 and 2022

		SDG 3c occupations workforce*			Other workers			Total		
**SNo**	**Country**	**Density per 10,000(2013)**	**Density per 10,000(2018)**	**Density per 10,000(2022)**	**Density per 10,000(2013)**	**Density per 10,000(2018)**	**Density per 10,000(2022)**	**Density per 10,000(2013)**	**Density per 10,000(2018)**	**Density per 10,000(2022)**
1	Algeria	2.93	41.89	49.17	0.05	57.07	81.72	2.98	98.96	130.90
2	Angola	0.01	19.34	22.16	0.23	16.09	16.30	0.24	35.43	38.46
3	Benin	7.69	4.83	8.28	14.61	9.79	10.45	22.30	14.62	18.73
4	Botswana	36.94	37.87	37.03	4.51	30.54	31.09	41.45	68.41	68.12
5	Burkina Faso	5.09	9.63	12.17	2.60	3.73	4.66	7.69	13.36	16.83
6	Burundi	7.06	9.16	8.31	0.04	32.73	32.68	7.11	41.90	40.99
7	Cameroon	10.32	5.80	8.34	-	6.72	2.99	10.32	12.51	11.33
8	Cape-Verde	13.17	29.17	62.96	141.90	92.38	41.40	155.07	121.55	104.36
9	Central African Republic	2.46	3.98	2.41	0.80	2.02	7.80	3.26	6.00	10.21
10	Chad	3.56	4.48	3.58	1.26	2.44	19.13	4.82	6.92	22.71
11	Comoros	6.46	19.85	22.33	12.02	15.65	9.01	18.49	35.50	31.33
12	Congo	9.47	12.80	13.05	10.20	6.08	7.28	19.66	18.87	20.33
13	Cote d'Ivoire	10.33	11.55	12.53	2.19	20.12	13.39	12.53	31.67	25.92
14	DRC	9.27	16.22	14.28	0.42	9.86	1.61	9.68	26.08	15.89
15	Equatorial Guinea	-	4.41	10.63	0.05	13.24	17.30	0.05	17.65	27.93
16	Eritrea	13.45	18.57	16.77	-	15.91	21.33	13.45	34.48	38.11
17	Eswatini	33.40	48.00	51.66	41.68	103.65	88.54	75.08	151.65	140.20
18	Ethiopia	0.71	9.79	18.58	0.05	14.40	15.25	0.77	24.19	33.83
19	Gabon	2.30	39.06	31.26	6.81	19.72	25.85	9.11	58.78	57.11
20	Gambia	14.02	9.34	8.15	4.72	10.71	26.02	18.74	20.05	34.17
21	Ghana	18.68	46.41	48.03	8.86	20.29	22.61	27.54	66.70	70.64
22	Guinea	7.49	8.90	6.64	6.06	14.41	13.05	13.55	23.31	19.69
23	Guinea-Bissau	7.27	10.89	12.88	2.92	32.02	30.76	10.19	42.91	43.64
24	Kenya	11.66	14.41	24.52	3.04	18.43	28.24	14.70	32.85	52.76
25	Lesotho	-	52.87	22.99	1.04	113.93	102.69	1.04	166.80	125.68
26	Liberia	2.20	22.91	13.44	4.60	28.36	43.56	6.80	51.27	57.00
27	Madagascar	4.45	5.46	4.76	0.07	16.43	17.21	4.52	21.89	21.97
28	Malawi	2.47	6.30	6.09	-	8.08	12.85	2.47	14.37	18.93
29	Mali	4.82	6.44	6.43	-	7.15	10.51	4.82	13.59	16.95
30	Mauritania	12.92	15.92	19.79	0.65	26.82	24.18	13.57	42.74	43.98
31	Mauritius	52.84	72.91	60.38	-	49.14	56.82	52.84	122.04	117.20
32	Mozambique	5.55	6.66	12.36	2.10	25.97	42.25	7.65	32.63	54.61
33	Namibia	18.95	64.85	72.50	-	39.79	32.85	18.95	104.65	105.35
34	Niger	2.94	2.73	2.36	3.52	1.59	2.60	6.45	4.32	4.96
35	Nigeria	14.99	22.25	22.69	18.95	26.80	13.89	33.94	49.05	36.58
36	Rwanda	10.44	14.19	13.40	2.41	39.64	46.22	12.85	53.83	59.62
37	Sao Tome and Principe	27.66	26.45	26.97	1.45	52.28	36.42	29.11	78.73	63.39
38	Senegal	4.56	7.02	6.52	3.63	21.99	17.28	8.19	29.00	23.80
39	Seychelles	54.80	195.92	242.01	-	50.73	54.45	54.80	246.65	296.47
40	SierraLeone	11.91	9.31	23.53	6.54	2.75	21.94	18.45	12.06	45.48
41	South Africa	16.72	61.78	78.19	4.36	36.78	31.95	21.08	98.56	110.14
42	South Sudan	-	6.17	7.90	-	9.74	11.81	-	15.91	19.70
43	Tanzania	4.75	6.78	7.61	3.05	12.00	21.08	7.80	18.77	28.68
44	Togo	3.56	5.14	6.30	0.90	27.90	23.93	4.46	33.04	30.24
45	Uganda	14.74	18.77	25.68	58.88	45.98	45.26	73.62	64.75	70.94
46	Zambia	2.81	17.26	34.96	3.58	18.15	20.98	6.39	35.41	55.94

		SDG 3c occupations workforce*			Other workers			Total		
**SNo**	**Country**	**Density per 10,000(2013)**	**Density per 10,000(2018)**	**Density per 10,000(2022)**	**Density per 10,000(2013)**	**Density per 10,000(2018)**	**Density per 10,000(2022)**	**Density per 10,000(2013)**	**Density per 10,000(2018)**	**Density per 10,000(2022)**
47	Zimbabwe	15.60	23.67	38.15	11.67	24.48	25.90	27.27	48.15	64.04
48	**WHO AFRICAN REGION**	**11.14**	**23.58**	**26.82**	**8.35**	**26.69**	**27.34**	**19.49**	**50.27**	**54.17**

- Indicates NO DATA

Appendix Table 26: The stock and density of doctors, nurses and midwives between 2013 and 2022

		Doctors Nurses and Midwives					
Sno	Country	2013	2018	2022	Density per 10,000 (2013)	Density per 10,000 (2018)	Density per 10,000 (2022)
1	Algeria	11,139	139,821	189,358	2.93	33.35	42.17
2	Angola	30	54,113	75,443	0.01	17.30	21.20
3	Benin	5,823	5,373	10,556	5.65	4.50	7.91
4	Botswana	8,136	8,570	9,020	36.69	34.96	34.29
5	Burkina Faso	8,590	18,578	26,578	4.87	9.11	11.72
6	Burundi	7,162	10,400	10,566	7.06	9.05	8.20
7	Cabo Verde	711	1,247	3,612	13.17	21.83	60.90
8	Cameroon	20,923	13,874	21,906	9.67	5.53	7.85
9	Central African Republic	1,160	1,974	1,332	2.42	3.87	2.39
10	Chad	4,630	6,538	5,798	3.50	4.19	3.27
11	Comoros	418	1,454	1,725	5.98	18.73	20.61
12	Congo	4,571	6,694	7,657	9.47	12.30	12.83
13	Cote d'Ivoire	17,871	26,225	32,978	7.95	10.29	11.71
14	Democratic Republic of the Congo	67,786	138,973	139,645	9.23	15.96	14.10
15	Equatorial Guinea		652	1,676	0.00	4.34	10.01
16	Eritrea	4,434	5,296	5,373	13.45	15.37	14.58
17	Eswatini	3,735	4,998	5,943	33.40	43.07	49.46
18	Ethiopia	6,925	89,196	189,544	0.71	8.03	15.36
19	Gabon	437	8,186	6,937	2.30	37.34	29.04
20	Gambia	2,858	2,116	2,082	13.45	8.65	7.70
21	Ghana	51,421	140,055	152,812	18.68	45.37	45.65
22	Guinea	8,133	10,848	8,857	7.36	8.64	6.39
23	Guinea-Bissau	1,234	1,999	2,607	7.27	10.38	12.38
24	Kenya	48,974	67,943	122,588	10.93	13.60	22.69
25	Lesotho		10,643	4,551	0.00	48.42	19.74
26	Liberia	974	10,108	6,367	2.20	20.67	12.01
27	Madagascar	10,361	13,784	13,784	4.39	5.13	4.65
28	Malawi	3,793	10,717	11,312	2.37	5.83	5.54
29	Mali	8,189	11,326	12,649	4.82	5.68	5.60
30	Mauritania	4,836	6,547	9,116	12.92	15.33	19.25
31	Mauritius	6,009	7,948	7,000	46.56	61.36	53.86
32	Mozambique	12,149	16,647	36,271	4.81	5.66	11.00
33	Namibia	4,110	14,406	17,172	18.64	59.88	66.90
34	Niger	5,396	6,078	6,096	2.89	2.69	2.33
35	Nigeria	241,954	385,486	453,649	13.85	19.43	20.76
36	Rwanda	11,351	16,464	17,139	10.22	13.14	12.44
37	Sao Tome and Principe	536	516	570	27.66	24.42	25.08
38	Senegal	5,628	10,475	10,579	4.14	6.73	6.11
39	Seychelles	512	1,670	2,284	52.94	161.98	213.25
40	Sierra Leone	8,293	6,714	19,043	11.91	8.54	22.13
41	South Africa	58,722	330,961	445,282	10.90	57.72	74.35
42	South Sudan		5,849	8,006	0.00	5.63	7.34
43	Tanzania	22,281	34,825	40,633	4.52	5.99	6.20
44	Togo	2,497	3,862	5,224	3.51	4.80	5.90
45	Uganda	50,519	77,330	116,206	14.32	18.63	24.59
46	Zambia	2,773	28,199	65,708	1.82	15.81	32.82
47	Zimbabwe	19,836	31,754	59,270	14.63	21.10	36.32
	**WHO AFRICAN REGION**	**767,820**	**1,807,432**	**2,402,504**	**10.56**	**21.07**	**24.69**

- Indicates NO DATA
